# C–H-Bond Activation and Isoprene Polymerization Studies Applying Pentamethylcyclopentadienyl-Supported Rare-Earth-Metal Bis(Tetramethylaluminate) and Dimethyl Complexes

**DOI:** 10.3390/molecules24203703

**Published:** 2019-10-15

**Authors:** Christoph O. Hollfelder, Melanie Meermann-Zimmermann, Georgios Spiridopoulos, Daniel Werner, Karl W. Törnroos, Cäcilia Maichle-Mössmer, Reiner Anwander

**Affiliations:** 1 Institute of Inorganic Chemistry, University of Tübingen, D-72076 Tübingen, Germany; christoph.hollfelder@anorg.uni-tuebingen.de (C.O.H.); melanie@meermann.net (M.M.-Z.); georgios.spiridopoulos@student.uni-tuebingen.de (G.S.); daniel.werner.07@gmail.com (D.W.); caecilia.maichle-moessmer@uni-tuebingen.de (C.M.-M.); 2 Department of Chemistry, University of Bergen, N-5007 Bergen, Norway; Karl.Tornroos@uib.no

**Keywords:** lanthanides, methyl, 1,3-diene polymerization, cluster, C-H-bond activation

## Abstract

As previously shown for lutetium and yttrium, 1,2,3,4,5-pentamethylcyclopentadienyl (C_5_Me_5_ = Cp*)-bearing rare-earth metal dimethyl half-sandwich complexes [Cp*LnMe_2_]_3_ are now also accessible for holmium, dysprosium, and terbium via tetramethylaluminato cleavage of [Cp*Ln(AlMe_4_)_2_] with diethyl ether (Ho, Dy) and tert-butyl methyl ether (TBME) (Tb). C–H-bond activation and ligand redistribution reactions are observed in case of terbium and are dominant for the next larger-sized gadolinium, as evidenced by the formation of mixed methyl/methylidene clusters [(Cp*Ln)_5_(CH_2_)(Me)_8_] and metallocene dimers [Cp*_2_Ln(AlMe_4_)]_2_ (Ln = Tb, Gd). Applying TBME as a “cleaving” reagent can result in both TBME deprotonation and ether cleavage, as shown for the formation of the 24-membered macrocycle [(Cp*Gd)_2_(Me)(CH_2_O*t*Bu)_2_(AlMe_4_)]_4_ or monolanthanum complex [Cp*La(AlMe_4_){Me_3_Al(CH_2_)O*t*Bu}] and monoyttrium complex [Cp*Y(AlMe_4_)(Me_3_AlO*t*Bu)], respectively. Complexes [Cp*Ln(AlMe_4_)_2_] (Ln = Ho, Dy, Tb, Gd) and [Cp*LnMe_2_]_3_ (Ln = Ho, Dy) are applied in isoprene and 1,3-butadiene polymerization, upon activation with borates [Ph_3_C][B(C_6_F_5_)_4_] and [PhNHMe_2_][B(C_6_F_5_)_4_], as well as borane B(C_6_F_5_)_3_. The trans-directing effect of AlMe_3_ in the binary systems [Cp*Ln(AlMe_4_)_2_]/borate is revealed and further corroborated by the fabrication of high-cis-1,4 polybutadiene (97%) with “aluminum-free” [Cp*DyMe_2_]_3_/[Ph_3_C][B(C_6_F_5_)_4_]. The formation of multimetallic active species is supported by the polymerization activity of pre-isolated cluster [(Cp*Ho)_3_Me_4_(CH_2_)(thf)_2_].

## 1. Introduction

The lanthanum complex [Cp*La{CH(SiMe_3_)_2_}_2_] (Cp* = C_5_Me_5_) reported by Schaverien and coworkers featured the first fully characterized discrete half-sandwich rare-earth metal bis(hydrocarbyl) complex, including an X-ray structure analysis [1]. Notwithstanding, polymerization-related research of this class of compounds has gained considerable momentum only 15 years later through the discovery by Hou et al., that cationized [(C_5_Me_4_SiMe_3_)Y(CH_2_SiMe_3_)(thf)][B(C_6_F_5_)_4_] acts as a highly efficient catalyst for the syndiospecific polymerization of styrene (>99% syndio; *M*_w_/*M*_n_ = 1.39) [2,3,4,5,6,7,8,9,10]. We have embarked on half-sandwich hydrocarbyl complexes of the type [Cp*Ln(AlMe_4_)_2_] as catalyst precursors for 1,3-diene polymerization [11]. It was revealed that upon cationization with perfluorinated borates/borane co-catalysts, such bis(tetramethylaluminate) complexes display highly efficient catalysts for living polymerization, giving access to gutta-percha type polymers in case of large rare-earth metal centers, as evidenced by the binary system [Cp*La(AlMe_4_)_2_]/B(C_6_F_5_)_3_ (trans-1,4 content: 99.5%, *M*_w_/*M*_n_ = 1.18) [12,13,14]. A major advantage of complexes [Cp*Ln(AlMe_4_)_2_] over other half-sandwich di(silyl)alkyl derivatives is their feasibility for the entire Ln(III) size range, enabling comprehensive investigations into the notoriously-known Ln(III) size-dependent polymerization performance [15,16,17,18,19,20,21,22]. Moreover, we have previously shown that for the smaller-sized Ln(III), a donor-induced tetramethylaluminato cleavage can be applied to generate the rare-earth metal dimethyl trimers [Cp*LnMe_2_]_3_ (Ln = Y, Lu) [23]. Crucially, the polymerization properties of the latter “aluminum-free” precatalysts showed that the isoprene polymerization is focused on a vinylic addition of the monomer, while in case of the slightly less bulky 1,3-butadiene a high cis-selectivity was observed (e.g., [Cp*YMe_2_]_3_/[PhNHMe_2_][B(C_6_F_5_)_4_]: cis-1,4 content: 95.2%, *M*_w_/*M*_n_ = 1.20) [24]. The latter study also provided insights into the active multimetallic polymerization species by ^1^H diffusion ordered spectroscopy (^1^H DOSY) and the solid-state structures of their degradation products, including methylidene clusters [(Cp*_3_Y_3_Me_4_(CH_2_)(thf)_2_] and [Cp*_6_Y_6_Me_4_(CH_2_)_4_] [24]. Given the importance of the Ln(III) size in 1,3-diene polymerization [25,26,27,28] and said advantages of our tetramethylaluminate approach, we herein present a more comprehensive picture thereof, giving further consideration to (a) the feasibility of “aluminum-free” [Cp*LnMe_2_]_3_ with larger ionic radii than Y(III); (b) potential competing “degradation” pathways, and (c) the application of defined isolable species in the polymerization of 1,3-dienes.

## 2. Results and Discussion

**Scheme 1:**
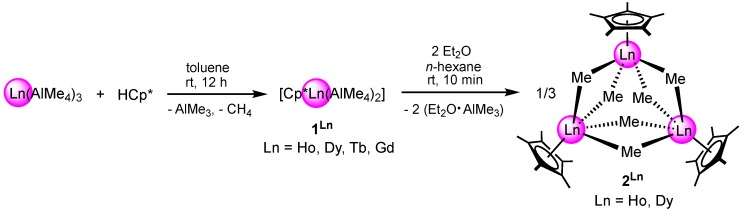
Protonolytic synthesis of [Cp*Ln(AlMe_4_)_2_] (**1^Ln^**) from the homoleptic tetramethylaluminates and subsequent donor-induced methylaluminato cleavage to generate [Cp*LnMe_2_]_3_ (**2^Ln^**).

The initial synthesis sequence for [Cp*LnMe_2_]_3_ (**2^Ln^**) of lanthanides with ionic radii larger than that of yttrium (0.90 Å for a coordination number of 6) [29,30] starts from the respective homoleptic tetramethylaluminates [Ln(AlMe_4_)_3_] [31,32,33,34,35,36,37]. A protonolysis protocol is then supposed to give access to the respective pentamethylcyclopentadienyl bis(alkylaluminate) complexes [Cp*Ln(AlMe_4_)_2_] (**1^Ln^**, Scheme 1) [12,13,14,38,39,40,41,42,43] which can undergo methlyaluminato cleavage by the addition of an ether (e.g., diethyl ether) [44] to form the respective dimethyl trimers **2^Ln^** [4,5,6,24,45]. Having previously established the respective chemistry of the rare-earth metals lanthanum, neodymium, yttrium [12,13,39] and lutetium [11,24] the current study accounts for the elements holmium, dysprosium, terbium, and gadolinium, thus closing the Ln(III) size gap between neodymium and yttrium by skipping the radioactive (Pm) and redox-active elements (Sm, Eu). Since most of the Ln(III) under study display enhanced paramagnetism, NMR-spectroscopic investigations have been restricted for reasons of limited information retrieval of relevance for any catalytic performance.

It will be revealed that in case of lanthanides larger than dysprosium, C–H-bond activation prevails upon alkylaluminato cleavage. This is known to occur with the yttrium analogue as well, when the cleavage reaction is performed above 40 °C, either in *n*-hexane or toluene, or only one equivalent of diethyl ether is applied [23]. Therefore, such cleavage reactions were also performed by applying tert-butyl methyl ether (TBME), and the products were investigated crystallographically.

### 2.1. Pentamethylcyclopentadienyl Lanthanide Bis(tetramethylaluminate) Compelxes

**Figure 1:**
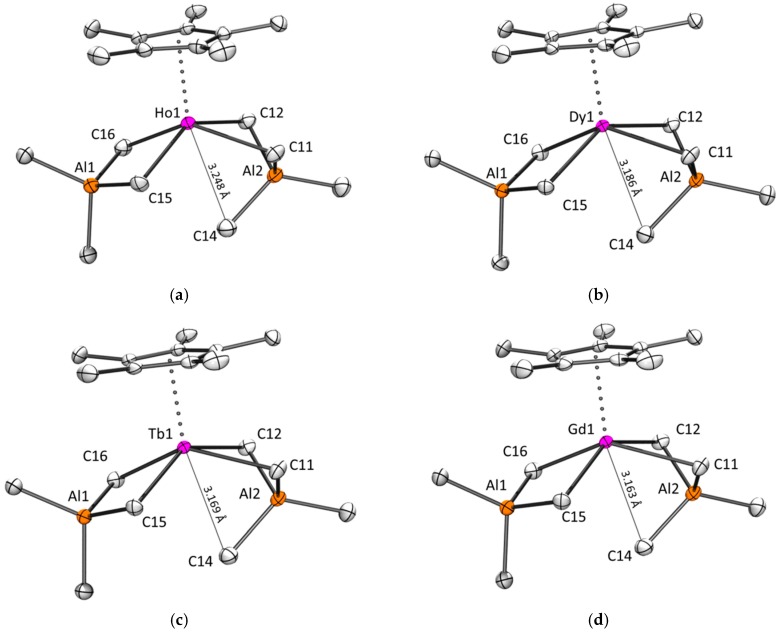
ORTEP view of the crystal structures of [Cp*Ln(AlMe_4_)_2_] (**1^Ln^**, Ln = Ho (**a**), Dy (**b**), Tb (**c**) and Gd (**d**)). Atomic displacement parameters are set at the 50% probability level. Hydrogen atoms are omitted for clarity. See Table 1 for significant bond lengths and angles.

As known for lanthanum [13], neodymium [39], samarium [43], yttrium [40], ytterbium [43], and lutetium [11,42], the pentamethylcyclopentadienyl bis(alkylaluminate) complexes **1^Ln^** of holmium, dysprosium, terbium, and gadolinium are accessible via the protonolysis protocol as shown in Scheme 1. Reacting pentamethylcyclopentadiene with the respective homoleptic [Ln(AlMe_4_)_3_] in toluene at ambient temperature gave virtually quantitative yields of [Cp*Ln(AlMe_4_)_2_] (**1^Ln^**). Single crystals suitable for X-ray diffraction analysis could be obtained by recrystallization from *n*-hexane (Figure 1a–d). Selected bond lengths and angles of **1^Ln^** are listed in Table 1.

In contrast to the dimethyl trimers (**2^Ln^**, Table 3, vide infra), the bis(alkylaluminate)s **1^Ln^** display clear tendencies to decreasing angles (C^Al1^–Ln–C^Al1^) and elongated bonds (Ln–C^Al^, Ln–Ct) with increasing ionic radii of the lanthanide center. Due to the monomeric nature of these complexes, the absence of cluster constraints allows the full display of the variation of the ionic radius of the lanthanide center (lanthanide contraction). In all cases, one of the two tetramethylaluminato moieties is tucked in, so that a third methyl unit of this tetramethylaluminato ligand is in relatively close proximity to the lanthanide center, as indicated in Figure 1. This gets more and more favored for larger ionic radii, which results in decreasing Ln---C14 distances (Table 1) and an increasing torsion angle. The tuck-in behavior clearly documents the influence of the rare-earth metal size on the ligand environment and is key to understand the polymerization behavior (vide infra). The results obtained in this study fit well to the series of data gained in previous studies for the other rare-earth metals [11,38,39].

**Figure 2:**
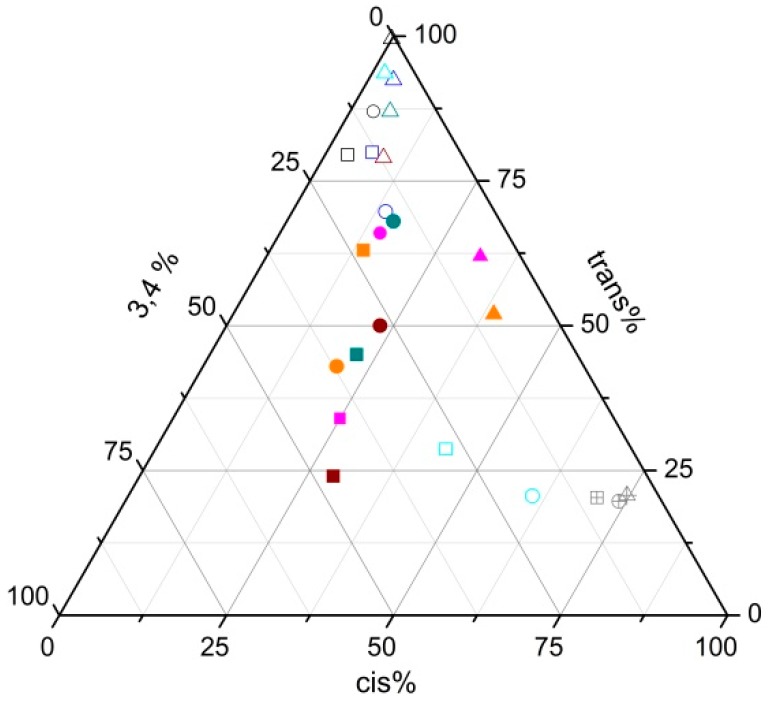
Ternary plot of the microstructures of the polyisoprenes obtained from [Cp*Ln(AlMe_4_)_2_] (**1^Ln^**). Polymers produced applying cocatalyst (**A**) are represented by circles, those produced with cocatalyst (**B**) by squares and products of activations with cocatalyst (**C**) are represented by triangles. Solid symbols mark reaction times of 1 h, hollow symbols mark reaction times of 24 h and hollow and crossed symbols mark reaction times of 15 min. The metal centers of the precatalyst is color-coded as follows: black (La), blue (Nd), teal (Gd), pink (Tb), orange (Dy), brown (Ho), cyan (Y), and grey (Lu). Data of the La, Nd, and Y analogues are shown for comparison [13].

The new complexes **1^Ln^** have been tested in the polymerization of isoprene (Table 2 and Figure 2). Roughly, for polymerizations cocatalyzed by perfluorinated borates [Ph_3_C][B(C_6_F_5_)_4_] (**A**) and [PhNHMe_2_][B(C_6_F_5_)_4_] (**B**) the previously noticed decrease of trans-selectivity with decreasing ion size was observed [13,14,42]. For the smaller lanthanides (Ln *<* Ho) relatively unselective microstructures with a slight tendency to 1,4-cis focused monomer addition are detected, while a trend toward 1,4-trans-selectivity occurred with increasing ionic radii of the lanthanide (Figure 2). In the process of activation by cationization, AlMe*_3_* is likely to be set free with precatalysts **A** and **B**. Trimethylaluminum is known to cause trans-shifts of microstructures, when added to catalytically active mixtures in 1,3-diene polymerization [24] (refs. in [46,47] serve as examples for trans-shifting behavior, due to exposition of the catalytically active species to free AlMe_3_; AlR_3_ with R > Me, also tested in the studies of refs [46,47], are known not to show microstructure alternating properties [24,48]. Moreover, if AlMe_3_ is not released upon catalyst activation, a 1,4-cis-diriging effect on the microstructure can be observed due to the formation of a bimetalic (Ln/Al) active species [49]). Although it has not been pointed out so far, to the best of our knowledge, the actual position of the equilibrium between AlMe_3_ coordination and release is a direct result of the accessible space at the rare-earth-metal center, likely exerting the trans-shifting influence [46,47,50].

As exceptions, polymers produced by the dysprosium and yttrium systems (Table 2, runs 14–15 and 21–22) should be further commented on. Next to the microstructures not being fully in line with the observed general trend, the low molecular weight average observed when cocatalyst **B** is applied to **1^Dy^** (Table 2, run 15) stands out slightly implying a high initiation efficiency. As yttrium and holmium are almost identical in ion size, much more similar polymers would be expected for these two metal centers. The observed difference implies the possibility of a change in selectivity over the reaction time as the yttrium system was reacted over 24 h (Table 2, runs 21 and 22), while the holmium-based polymerizations were quenched after 1 h in order to obtain an impression of the polymerization rate (Table 2, runs 17 and 18). Verification of this theory by applying the yttrium system with a polymerization time of 1 h is a subject to future research.

It is surprising as well that the small lutetium polymerizes much faster than the other systems, producing quantitative yields even after 15 min. As the obtained polymers are relatively similar for the smallest lanthanide element independent of the employed cocatalyst (Table 2, runs 24–26), in contrast to all other systems, a different active species has to be considered. Figure 2 also shows a high agglomeration of hollow symbols, marking the polymerization runs for 24 h, in the high-trans area of Figure 2. This is most significant for polymers produced in the presence of borane [B(C_6_F_5_)_3_] (**C**) as a cocatalyst (shown as triangles in Figure 2). The throughout high trans-contents applying cocatalyst **C** can be reasoned, regarding earlier reactions of the cocatalyst with alkylaluminates. Accordingly, the likely formation of perfluoroaryl alkylaluminate anionic species of the type {{Cp*Ln[(*µ*-Me)_2_AlMe(C_6_F_5_)]} ^+^ [Me_2_Al(C_6_F_5_)_2_]^-₋^}_2_ [12] features active species that differ greatly from those obtained by the application of cocatalysts **A** and **B**. Such an alternation of the active species, taking place with a much lower reaction rate than the chain propagation, explains why the microstructure produced by **1^Dy^**/**C** after 1 h (Table 2, run 16) has a much lower trans-content than the polymerizations by the other systems, which reacted for 24 h. Once again, verification by polymerization runs applying **1^Dy^** for 24 h is a subject to research. The results for Ln = Lu (Table 2, run 26), obtained with the much faster polymerization, observed in general for **1^Lu^**, would not interfere with the presence of this alternation reaction. 

### 2.2. Ether-Promoted Methylaluminato Cleavage Reactions of [Cp*Ln(AlMe_4_)_2_] (**1^Ln^**)

#### 2.2.1. Holmium and Dysprosium

**Figure 3:**
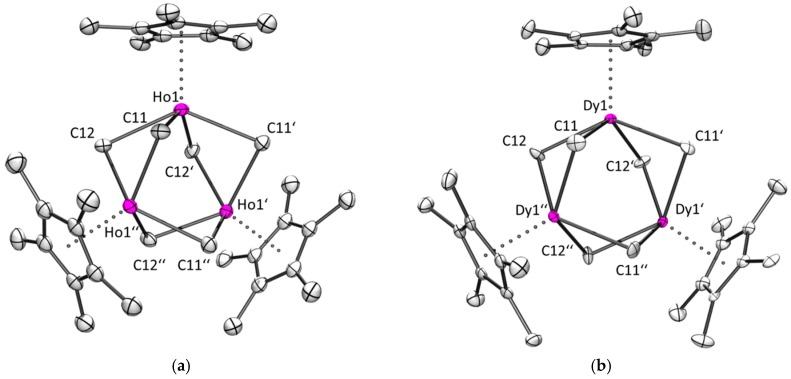
ORTEP view of the crystal structures of [Cp*HoMe_2_]_3_ (**2^Ho^**, (**a**)) and [Cp*DyMe_2_]_3_ (**2^Dy^**, (**b**)). Atomic displacement parameters are set at the 50% probability level. Hydrogen atoms are omitted for clarity. See Table 3 for significant bond lengths and angles.

Following the tetramethylaluminate cleavage protocol [44], which has been successfully applied in the case of yttrium and lutetium [24], trimeric [Cp*LnMe*_2_*]*_3_* (**2^Ln^***)* were obtained for holmium and dysprosium as well (Scheme 1). While the cleavage with diethyl ether was quantitative with Ln = Ho, a yield of only 19% could be achieved for Ln = Dy at ambient temperature. Strikingly, the dysprosium-derived reaction mixture turned into a much deeper yellow color than the pale yellow typical for dysprosium compounds. Empirically, this color change has shown to be an indication of ongoing C-H-bond activation in such cleavage reactions. Here, as pointed out earlier [24], multiple products are formed, which can explain why none of the degradation products could be isolated in the case of dysprosium. Single crystals of **2^Ho^** and **2^Dy^** suitable for X-ray diffraction analyses could be obtained from recrystallization in *n*-hexane (Figure 3a,b). Relevant bond lengths and angles are given in Table 3. Interestingly, the comparison of selected bond lengths and angles of the dimethyl trimers **2^Ln^** does not show any clear dependency on the ionic radius of the respective lanthanide center. It is assumed that the geometry of the cyclic trimer simply does not provide the freedom for ideal ligand arrangement. This might also cause the reduced stability of the trimers, observed with increasing Ln(III) ionic radius, which then results in a higher tendency to undergo side reactions, e.g., C-H-bond activation reactions or ligand redistribution.

**Figure 4:**
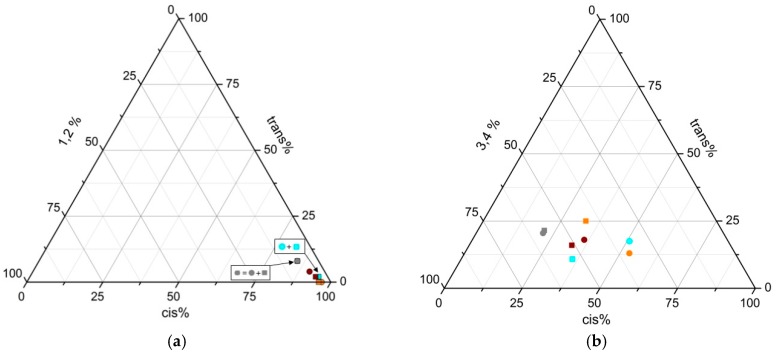
Ternary plot of the microstructures of the poly-1,3-butadienes (**a**) and polyisoprenes (**b**) obtained from of [Cp*LnMe_2_]_3_ (**2^Ln^**). Polymers produced applying cocatalyst **A** are represented by circles, those produced with cocatalyst **B** by squares. The metal centers of the precatalyst are color-coded as follows: orange (Dy), brown (Ho), cyan (Y), and grey (Lu). Data for the reactions applying the Y and Lu analogue precatalysts are shown for comparison [24].

Both the holmium and the dysprosium dimethyl trimers have been tested in the polymerization of 1,3-dienes. Due to the similarity of holmium and yttrium in ion size, the reactivity and polymerization outcome with 1,3-butadiene differ only slightly (Table 4, runs 29–32, Figure 4a, [24]) regarding the microstructure. The polymerizations of isoprene with the binary system **2^Ho^**/**B** revealed a similar analogy (Table 5, runs 38 and 40). Only when cocatalyst **A** ([Ph_3_C][B(C_6_F_5_)_4_]) was applied, the microstructure differs. An active species, behaving similar to that produced by **B**, is formed in the case of Ln = Ho. In contrast to **2^Y^**/**A** (Table 5, Figure 4b, [24]), this leads reproducibly to only little variance between the **A** and **B** runs (Table 5, runs 37 and 38).

Regarding the chain properties, once again the polymers produced by **2^Ho^**/**A** differ from **2^Y^**/**A**, since with **2^Ho^**, no indication of a second, underlying crosslinking reactivity was found [24]. Monomers, yields, polydispersity index (PDI), and molecular weight averages imply a much more uniform and simultaneous activation for holmium. However, in the case of holmium, it should be pointed out that except for the polybutadiene obtained from the system **2^Ho^**/**A**, all observed molecular weight averages are high enough to imply ratios of chains per catalytic center far smaller than 1. Therefore, the active species are supposed to consist of more than one holmium center, as observed for yttrium [24].

1,3-Butadiene polymerization applying **2^Dy^** (Table 4, runs 27 and 28) led to highly cis-selective microstructures (97%), as observed for holmium and yttrium as well. Moreover, high yields were obtained, which were not achieved in the case of yttrium and only for cocatalyst **A** with holmium. Next to these, differences to the smaller metals occurred regarding the chain properties. For the binary systems **2^Dy^**/**A** and **2^Dy^**/**B**, the observed molecular weights implies that there are ratios of chains per lanthanide center much smaller than 1. This means that in case of dysprosium, far less than every metal center grows a polymer chain independent of the selected cocatalyst. Therefore, active species with more than one lanthanide atom are considered likely here as well. The PDI appeared—although significantly increased compared to the smaller lanthanides—too low to ascribe the increased molecular weight averages to merely an initiation deficiency.

In case of the **2^Dy^**-promoted isoprene polymerization (Table 5, runs 35 and 36), the uniformity of the microstructure, as observed for holmium, is not found. While cocatalyst **B** produces a polymer in good analogy to those obtained from holmium; in case of cocatalyst **A**, much higher cis-content is obtained. This finding decisively corroborates the cis-preference of half-sandwich complexes in the absence of AlMe_3_ [46,47,50]. The comparably small molecular weight average of the polymer produced by **2^Dy^**/**A** is in the vague region of one chain per Dy center and a significantly increased PDI put in a much closer relation to the polymer produced by the system **2^Y^**/**A** (Table 5, run 38. [24]). Furthermore, THF insoluble parts of the polymer were observed, which is in agreement with the previous findings for the yttrium system as well. There, an underlying second mechanism likely causes crosslinking of the polymer chains [24].

#### 2.2.2. Terbium

**Scheme 2:**
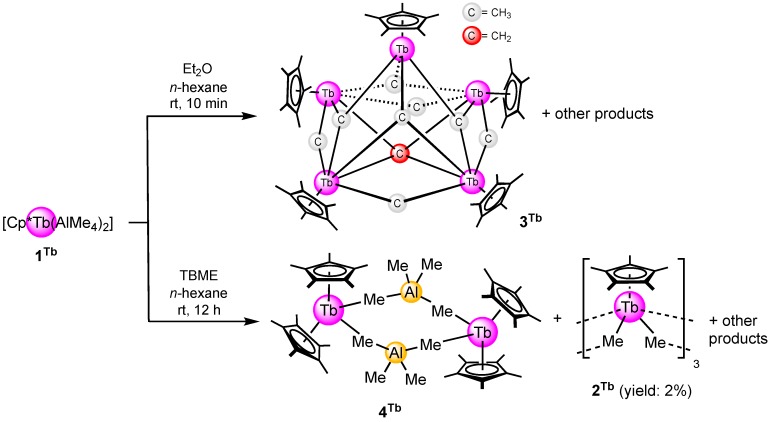
Diethyl ether-induced cleavage and C–H-bond activation of [Cp*Tb(AlMe_4_)_2_] (**1^Tb^**) yielding pyramidal complex (**3^Tb^**). Using tert-butyl methyl ether (TBME), ligand redistribution and the formation of metallocene alkylaluminate **4^Tb^** prevails; [Cp*TbMe_2_]_3_ (**2^Tb^**) is a side product in this reaction.

**Figure 5:**
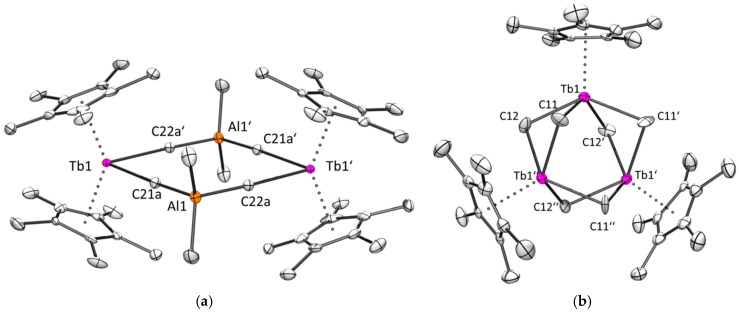
Panel (**a**): ORTEP view of the crystal structure of [Cp*_2_Tb(AlMe_4_)]_2_ (**4^Tb^**). Atomic displacement parameters are set at the 50% probability level. Hydrogen atoms and one molecule of *n*-hexane per asymmetric unit are omitted for clarity. See Table 6 for significant bond lengths and angles. Panel (**b**): ORTEP view of the crystal structure of [Cp*TbMe_2_]_3_ (**2^Tb^**). Atomic displacement parameters are set at the 50% probability level. Hydrogen atoms are omitted for clarity. See Table 3 for significant bond lengths and angles.

In case of terbium, the cleavage reaction applying diethyl ether produced a brown slurry (Scheme 2), which showed to be a mixture of several (in solution) non-isolable C-H-bond activation products. Only one of those, a pyramidal shaped cluster [(Cp*Tb)_5_(CH_2_)(Me)_8_] (**3^Tb^**), could be crystallized (vide infra). Applying TBME, an ether that does not possess “reactive” hydrogen atoms, which excludes one possible C-H-bond activation pathways, surprisingly led to the metallocene alkylaluminate [Cp*_2_Tb(AlMe_4_)]_2_ (**4^Tb^**). Complex **4^Tb^** crystallized from the reaction solution as the main product (Figure 5a). Complexes of this type were originally synthesized by Lappert [18], Watson [51], and Evans [52], and later on successfully applied in 1,3-diene polymerization by Kaita et al. [25,26]. Crystal structures were obtained for [Cp_2_YMe]_2_ [44], [Cp*_2_Y(AlMe_4_)]_2_ [51], [Cp*_2_Sm(AlMe_4_)]_2_ [52], and [Cp*_2_Ln][B(C_6_F_5_)_4_] (Ln = Pr, Nd) [25].

Such ligand redistribution, leading to the metallocene **4^Tb^** implies the existence of a second product formed from **1^Tb^** or in situ formed **2^Tb^**, which has served as a source for the second Cp* ligand, as the stoichiometry of terbium and the Cp* ligand deviates from the original one in **1^Tb^**. So far, this second product has not been identified and remains a subject to research. Instead, the originally intended dimethyl trimer **2^Tb^** was found to be a low-yielding byproduct of this reaction (2% crystallized yield). Single crystals of **2^Tb^** suitable for X-ray diffractometry could be obtained from recrystallization of the powder having formed during the reaction in *n*-hexane (Figure 5b). Selected bond lengths and angles can be found in Table 3.

**Figure 6:**
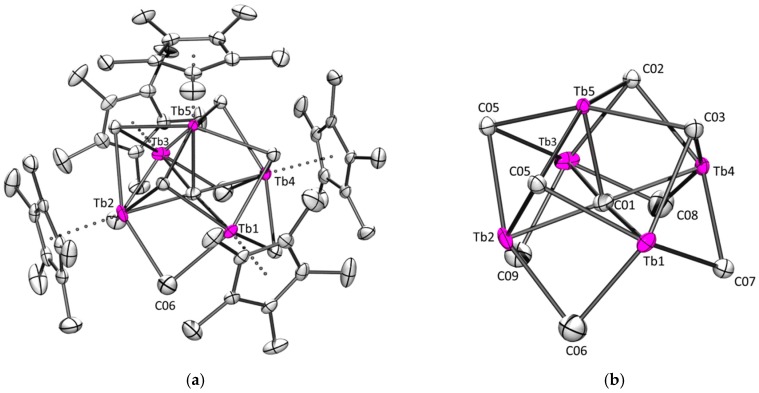
ORTEP view of the crystal structure (**a**) and detailed view on the bonding situation in the core structure (**b**) of [(Cp*Tb)_5_(CH_2_)(Me)_8_] (**3^Tb^**). Atomic displacement parameters are set at the 50% probability level. Hydrogen atoms and one molecule of *n*-hexane per asymmetric unit are omitted for clarity. See Table 7 for significant bond lengths and angles.

In the case of the originally performed cleavage reaction with diethyl ether, the methyl/methylidene cluster [(Cp*Tb)_5_(CH_2_)(Me)_8_] (**3^Tb^**) was obtained as the only isolable product with a yield of < 5%. It is a*ss*umed to be one of many formed C–H-bond activation products. Since it was possible to investigate this species crystallographically (and further structures of this motif of Gd as well, vide infra), further details on it are provided here. Complex **3^Tb^** contains five Cp*Tb fragments, that are arranged in a square pyramidal motif (Figure 6a). A CH*_2_^2−^* anion is located in the center of the basal face of the pyramid (Figure 6b). The other eight remaining negative charges are assigned to eight methyl moieties both bridging the four vertex terbium atoms of the pyramid base and capping its triangular faces. For further comparison, Ln_x_ methyl/methylidene clusters with x > 3 are rare [23,24,45,46,47] comprising [(Cp*Y)_4_(AlMe)_2_Me_8_(CH_2_)_2_] [23], [Cp’Ln(CH_2_)]_4_ (Ln = Tm, Lu) [45], [(Cp’Y)_4_(Cp*Ir)(CH_2_)Me(H)_3_(O)_2_] [53], [(Cp’Lu)_4_(CH_2_)_3_Me][B(C_6_F_5_)_4_] [54], and [Cp*_6_Y_6_Me_4_(CH_2_)_4_] [24], with Cp’ = C_5_Me_4_SiMe_3_.

**Figure 7:**
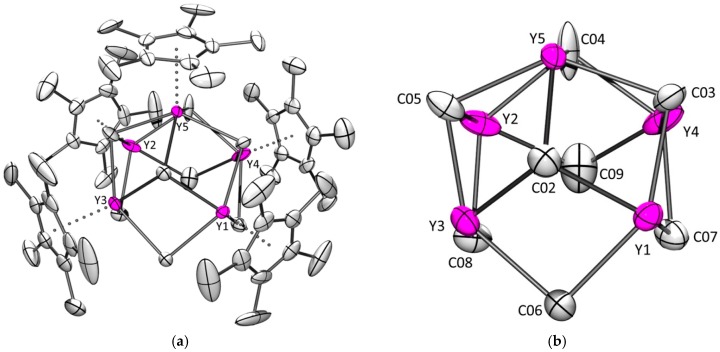
ORTEP view of the crystal structure (**a**) and detailed view on the bonding situation in the core structure (b) of [(Cp*Y)_5_(Me)_6_(CH_2_)_2_] (**5^Y^**). Atomic displacement parameters are set at the 30% (**a**) and the 50% (**b**) probability level. Hydrogen atoms and one molecule of toluene per asymmetric unit are omitted for clarity. See Table 7 for significant bond lengths and angles.

**Figure 8:**
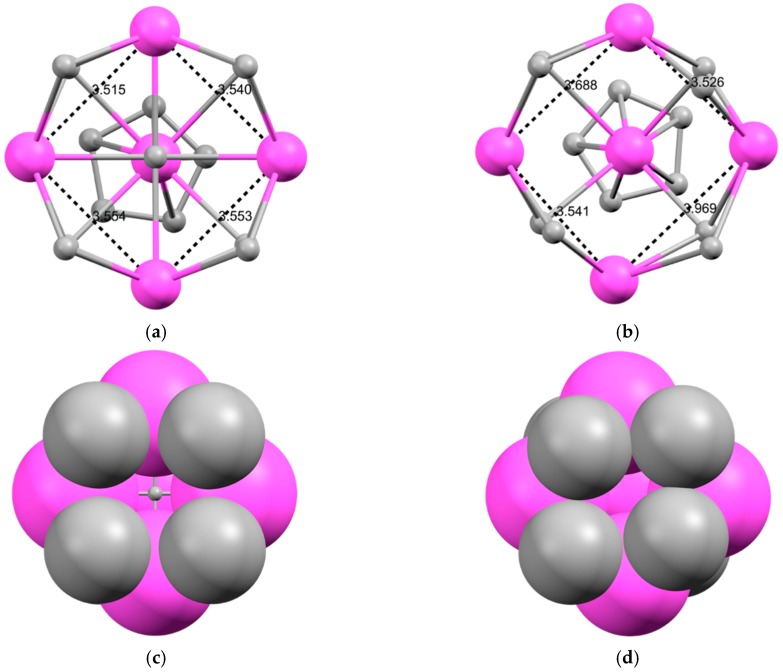
Comparison of the coordinative geometry in complexes **3^Gd^** ((**a**) and (**c**)) and **5^Y^** ((**b**) and (**d**)). In panels (**a**) and (**b**), distances (Å) and a geometry overview of the core structure (neglecting the Cp* anionic ligands, except for that binding to the apical Ln center) are given in a ball and stick model. Panels (**c**) and (**d**) focus on the geometry of the respective base tetragon of the pyramidal cluster in visualizing the space filling of the respective atoms. Their square and rhomboid shape can clearly be observed. In panel (**c**), the centering carbon atom of the methylene dianion is visible.

This structure of the pentametallic cluster **3^Tb^** differs significantly from the C-H-bond activation product found in a pyramidal pentametallic yttrium methyl/methylidene complex (**5^Y^**, Figure 7 and Table 7) through the existence of the central methylene dianion. It is proven not to be present in this position in the yttrium case. Besides that, the terbium and yttrium pyramid differ by the shape of the tetragonal face of the pyramid as well (Figure 8). In case of **5^Y^**, the pyramidal arrangement does not show rectangular vertices (Figure 8b) and the bridging methyl carbon atoms form a much more rhomboid-like tetragon (Figure 8d). No conclusive hydrogen atom assignment could be derived for the yttrium core, hence allowing for the speculation of a composition of **5^Y^** of the bis(methylidene) complex [(Cp*Y)_5_(Me)_6_(CH_2_)_2_] to compensate for the missing anionic charge. Complex **5^Y^** was obtained from reacting [Cp*YMe_2_]_3_ (**2^Y^**) with metallocene tetramethylaluminates [Cp*_2_Ln(AlMe_4_)]_2_ (Ln = Y, La) overnight at ambient temperature.

#### 2.2.3. Gadolinium

**Scheme 3:**
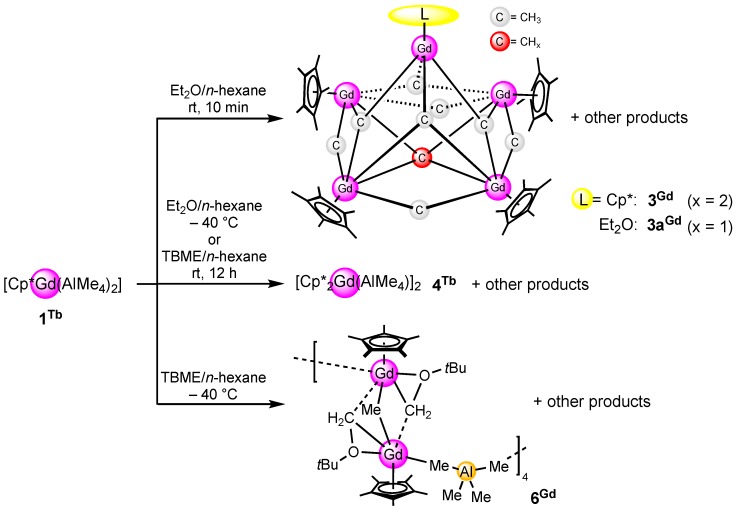
Attempted methylaluminato cleavage reaction of [Cp*Gd(AlMe_4_)_2_] (**1^Gd^**) with diethyl ether toward [Cp*GdMe_2_]_3_ (**2^Gd^**, not isolable). Pyramidal complex **3^Gd^** is the main product of this reaction, along with **3a^Gd^** resulting from ligand exchange. It is assumed, that putative **2^Gd^** is an intermediate en route to **3^Gd^**. Using TBME, the metallocene alkylaluminate **4^Gd^** is obtained by ligand redistribution. This product is also obtained when the activation with diethyl ether is performed at −40 °C. When the activation by TBME is performed at −40 °C, the octameric macrocycle **6^Gd^** was observed as a product.

Concluding from the results obtained from the synthesis approaches with terbium precursors, gadolinium—with its even larger ionic radius—should increasingly favor C–H-bond activation reactions. Therefore, similar but not identical behavior, as in case of terbium, was found (Scheme 3). Here, putative trimeric complex **2^Gd^** could not be detected/isolated for both ether reagents (diethyl ether and TBME) probed in the attempt to cleave the alkylaluminato moiety. Instead, once more, products of C–H-bond activation and ligand redistribution reactions were found.

**Figure 9:**
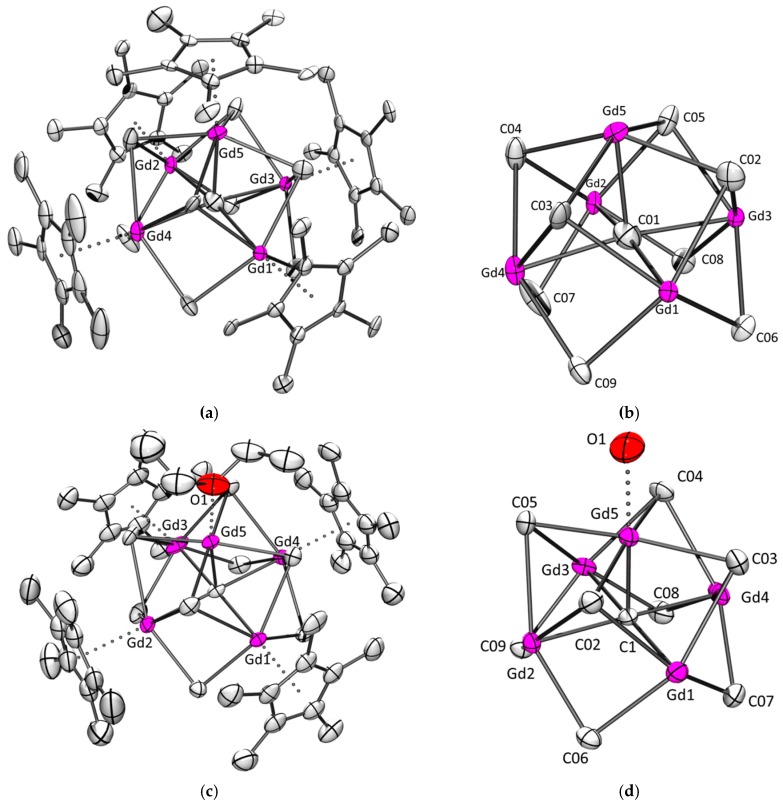
ORTEP view of the crystal structure (**a**) and detailed view of the bonding situation in the core structure (**b**) of [(Cp*Gd)_5_(CH_2_)(Me)_8_] (**3^Gd^**). Panels (**c**) and (**d**) show the structure and the bonding situation in [(Cp*Gd)_4_{(Et_2_O)Gd}(CH)(Me)_8_] (**3a^Gd^**) likewise. Atomic displacement parameters are set at the 50% probability level. Hydrogen atoms, and in the case of **3a^Gd^**, a second species of the complex and two molecules of *n*-hexane per asymmetric unit are omitted for clarity. See Table 7 for significant bond lengths and angles.

As in case of terbium, pyramidal pentametallic gadolinium structures (**3^Gd^** and **3a^Gd^**) could be isolated as products of the cleavage attempt of **1^Gd^** applying diethyl ether. Here, two different species of this type were found to crystallize next to each other. The apparently by far dominating amount of crystals revealed to be the analogous pyramidal C–H-bond activation product of [(Cp*Gd)_5_(CH_2_)(Me)_8_] (**3^Gd^**, Figure 9a,b) as found for terbium (Figure 6). Interestingly, in **3a^Gd^**, one Cp* ligand of **3^Gd^** is replaced by a diethyl ether donor ligand (Figure 9c,d). Therefore, the ion located in the center of the tetragonal face of the pyramid of **3a^Gd^** is assumed to be a methylidyne trianion to compensate for the missing anionic charge of the displaced Cp*. The cleavage of Cp* ligands seems unprecedented in this type of reactions and provides an indication of the possible nature of the second product of the metallocene alkylaluminate dimer (**4**) producing reactions (vide infra for Tb, vide supra for Gd). However, the yields of **4** seem to be too high to allow a second product that contains five lanthanide atoms per released Cp* ligand.

**Figure 10:**
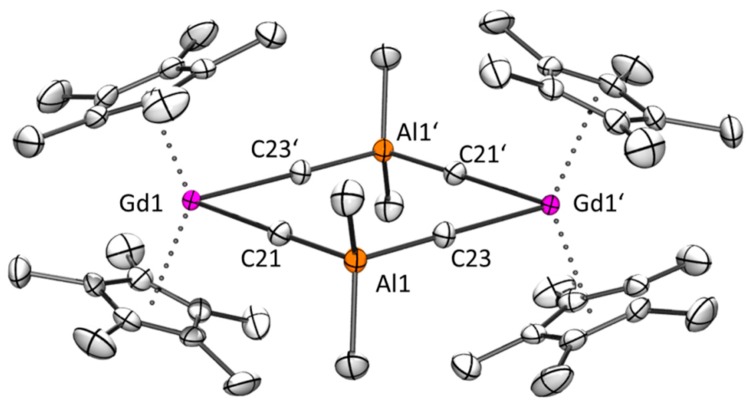
ORTEP view of the crystal structure of [Cp*_2_Gd(AlMe_4_)]_2_ (**4^Gd^**). Atomic displacement parameters are set at the 50% probability level. Hydrogen atoms and one molecule of *n*-hexane per asymmetric unit are omitted for clarity. See Table 6 for significant bond lengths and angles.

In order to avoid C–H-activation pathways, the methylaluminato cleavage of **1^Gd^** with diethyl ether was performed at lower reaction temperatures (e.g., −40 °C). However, the putative dimethyl trimers **2^Gd^** could not be isolated this way. Instead, the metallocene ligand-redistribution product [Cp*_2_Gd(AlMe_4_)]*_2_* (**4^Gd^**) was obtained (Figure 10). Paralleling the formation of the terbocene alkylaluminate **4^Tb^**, the gadolinium analogue **4^Gd^** was obtained as the main product of the attempted cleavage reaction with TBME as well, when the reaction was performed at ambient temperature. Comparing the selected bond lengths and angles for the known [Cp*_2_Ln(AlMe_4_)]_2_ (Table 6), indicates that only the bond lengths adjust to the ionic radii of the lanthanide centers, while the angles deviate only marginally. Therefore, the potential tension built up, even in cases of large lanthanides, appears to not be high enough to cause subsequent reactions via C–H-bond activation (e.g., by release of methane).

**Figure 11:**
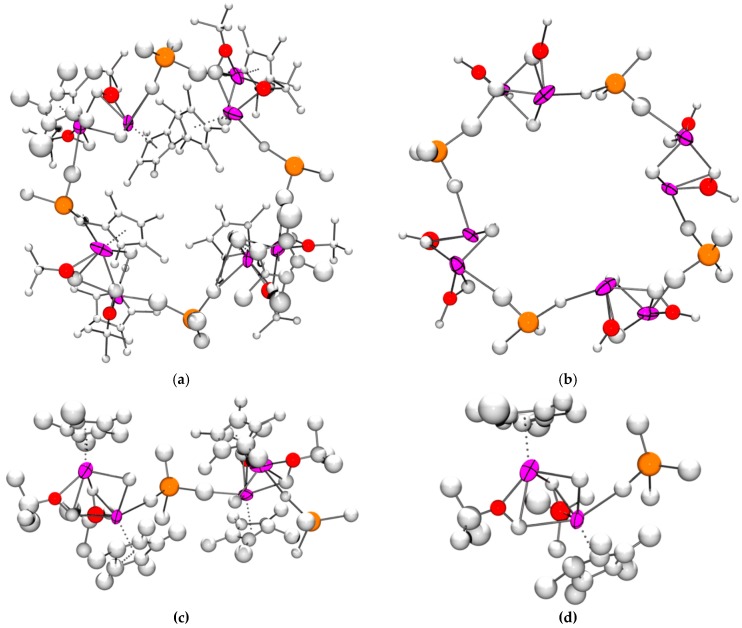
Connectivity overview of product **6^Gd^**, considered to be 24-membered macrocyclic compound [(Cp*Gd)_2_(Me)(CH_2_O*t*Bu)_2_(AlMe_4_)]_4_, of the reaction of [Cp*Gd(AlMe_4_)_2_] (**1^Gd^**) with TBME. In the ball and stick model, grey represent carbon atoms, magenta represent gadolinium atoms, orange represent aluminum and the red depict the location of oxygen atoms. Hydrogen atoms are omitted for clarity. Due to high R-values of the measurement, no further detailed figure is meaningful. While panel (**a**) gives an overview on the 24-membered macrocyclic complex, the further panels contain detailed views on the bridging/linking situation (**b**), the asymmetric Gd_4_Al_2_ unit (**c**), and the Gd_2_Al repetition unit (**d**).

Surprisingly, when TBME was applied as a cleaving agent at –40 °C, the dodecametallic ether activation product [(Cp*Gd)_2_(Me)(CH_2_O*t*Bu)_2_(AlMe_4_)]_4_ (**6^Gd^**) formed (Figure 11). Complex **6^Gd^** is arranged in form of a 24-membered macrocycle (Figure 11b) containing two symmetry-related dimeric Gd_4_Al_2_ entities (Figure 11c). Each of these are built up from two chemically identical Gd_2_Al parts (Figure 11d). In the latter units, the two gadolinium centers are bridged by one methyl group and two TBME units, activated at the former methyl moiety of the ether, which is necessary for charge balance. This macrocycle formed reproducibly, but due to the sheer size of the unit cell, structure elucidation by X-ray diffraction turned out to be rather challenging. Up to now, only connectivity pictures can be shown (Figure 11a–d).

#### 2.2.4. More about Yttrium and Lanthanum: Structural Snapshots

**Figure 12:**
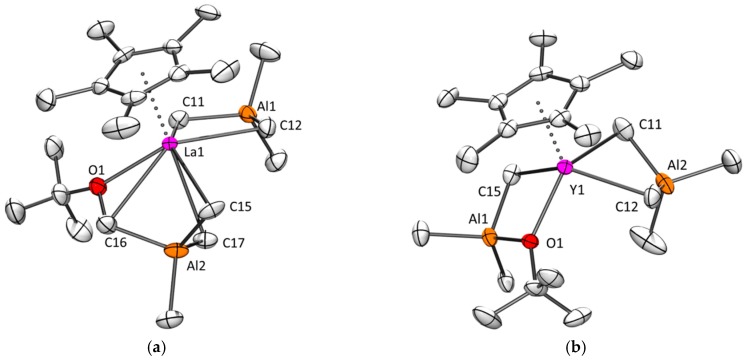
ORTEP view of the crystal structures of [Cp*La(AlMe_4_){Me_3_Al(CH_2_)O*t*Bu}] (**7^La^**, (**a**)) and of [Cp*Y(AlMe_4_)(Me_3_AlO*t*Bu)] (**8^Y^**, (**b**)). Atomic displacement parameters are set at the 50% probability level. Hydrogen atoms and in both cases a second species of the respective complex per asymmetric unit are omitted for clarity. Significant lengths (Å) and angles (°) for **7^La^**: La1–C^Cp*^: 2.781(5)–2.801(6); La1–Ct: 2.523; La1–C11/12: 2.787(6), 2.755(6); La1–C15: 2.956(5); La1–O1: 2.445(3); O1–C16: 1.478(5); Ct-La1-C11/12: 103.62, 111.49; Ct-La1-C15: 100.74; Ct-La1-O1: 106.06; O1-C16-Al2: 121.5(3). Significant lengths (Å) and angles (°) for **8^Y^**: Y1–C^Cp*^: 2.620(3)–2.656(4); Y1–Ct: 2.342; Y1–C11/12: 2.697(4)/2.624(3); Y1–C15: 2.586; Y1–O1: 2.247(2); Al1–O1: 1.858(2); Ct–Y1–C11/12: 107.84, 106.41; Ct–Y1–C15: 110.07; Ct–Y1–O1: 118.99.

The formation of half-sandwich methylidene and methine activation products in the presence of THF or diethyl ether has been examined previously and includes structurally characterized [Cp*_3_Ln_3_(µ-Cl)_3_(µ_3_-Cl)(µ_3_-CH_2_)(thf)_3_] (Ln = Y, La) [55], [Cp*_4_Y_4_(µ_2_-CH_3_)_2_{(CH_3_)Al(µ_2_-CH_3_)_2_}_4_(µ_3_-CH)_2_] [23], and [(Cp*_3_Y_3_Me_4_(CH_2_)(thf)_2_] [24]. Giving the distinct reactivity pathways of complexes **1^Ln^** triggered by different ethereal molecules, we treated [Cp*La(AlMe_4_)_2_] (**1^La^**) with TBME at −40 °C. The crystallized half-sandwich complex [Cp*La(AlMe_4_){Me_3_Al(CH_2_)O*t*Bu)}] (**7^La^**) bears an unprecedented tetradentate monoanionic ligand [Me_3_Al(CH_2_)O*t*Bu] formed by C–H-bond activation involving an alkylaluminato ligand and TBME under release of methane (Figure 12a). Interestingly, the second alkylaluminato moiety stays intact. Conducting the corresponding yttrium-reaction at ambient temperature led to complex [Cp*Y(AlMe_4_)(Me_3_AlO*t*Bu)] (**8^Y^**) featuring the bidentate monoanionic ligand [Me_3_AlO*t*Bu)]. A likely scenario for the formation of [Me_3_AlO*t*Bu]^-^ comprises an initial methylaluminato cleavage, followed by ether cleavage through a transient terminal [Ln–CH_3_] moiety, most likely under ethane release. Finally, the concomitantly formed Ln–O*t*Bu moiety reassembles with initially separated AlMe_3_ to [Me_3_AlO*t*Bu)] (Figure 12b). It is noteworthy that this ligand has been produced previously by the addition of AlMe_3_ to Ln(III) tert-butoxides [56,57]. Furthermore, applying TBME to [Cp*Y(AlMe_4_)_2_] (**1^Y^**) at −40 °C led to a cleavage reaction resulting in [Cp*YMe_2_]_3_ (**2^Y^**). When TBME was applied to the holmium analogue [Cp*Ho(AlMe_4_)_2_] (**1^Ho^**)—both at ambient temperature and at −40 °C—the trimeric dimethyl (**3^Ho^**) was obtained.

### 2.3. C–H-Bond Activation of [Cp*HoMe_2_]_3_ upon Reaction with THF

**Scheme 4:**
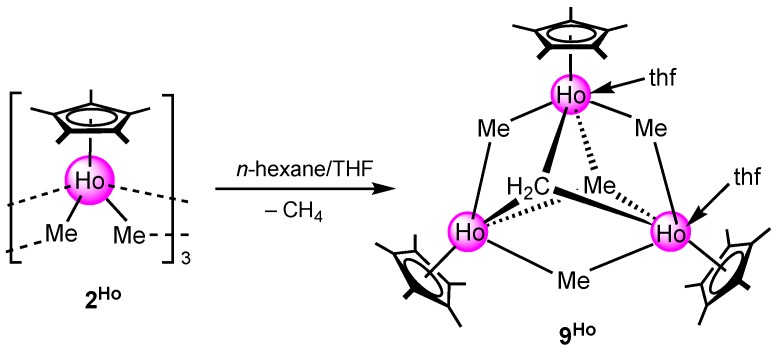
Synthesis of [(Cp*Ho)_3_Me_4_(CH_2_)(thf)_2_] (**9^Ho^**) from [Cp*HoMe_2_]_3_ (**2^Ho^**) in analogy to the reaction applying the respective yttrium compound [24].

**Figure 13:**
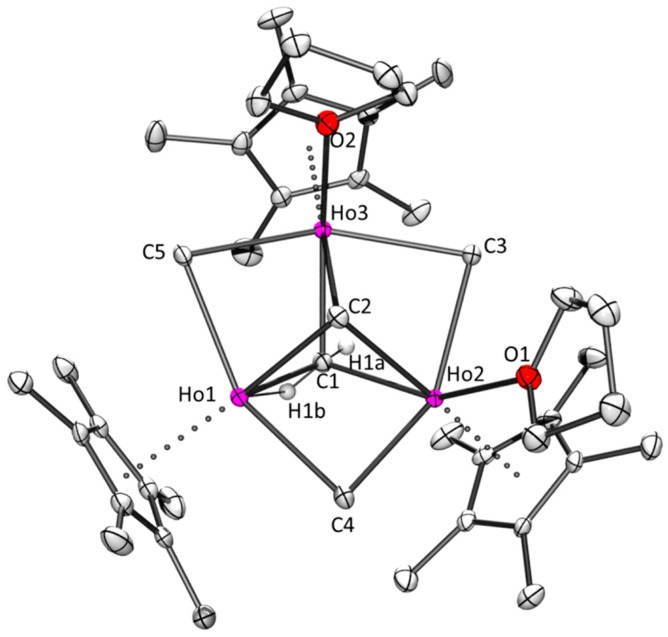
ORTEP view of the crystal structure of [(Cp*Ho)_3_Me_4_(CH_2_)(thf)_2_] (**9^Ho^**). Atomic displacement parameters are set at the 50% probability level. Hydrogen atoms are omitted for clarity except for H1a and H1b. Significant lengths (Å) and angles (°) for **9^Ho^**: Ho–C^Cp*^: 2.678(4)–2.713(4); Ho–Ct: 2.402 – 2.415; Ho–C3/4/5: 2.535(4)–2.582(5); Ho–C1: 2.275(4)–2.468(4); Ho–C2: 2.558(4)–2.764(4); Ho-O1/2: 2.571(3), 2.631(3); Ct–Ho–C3/4/5: 103.44–107.76; Ct–H–C1: 108.15–122.98; Ct–Ho–C2: 151.82–172.56; Ct–Ho–O1/2: 99.32, 100.24.

As for yttrium, the reaction of the dimethyl trimer **2^Ho^** with an excess of THF accomplished [(Cp*Ho)*_3_*Me*_4_*(CH*_2_*)(thf)*_2_*] (**9^Ho^**, Scheme 4 and Figure 13) as a stable C–H-bond activation product. Further activation of the methyl/methylidene cluster **9^Ho^** with cocatalyst **B** resulted only in a small amount of holmium centers active in the polymerization of isoprene (as likewise observed in the yttrium study [24]). However, with cocatalyst **A**, two main active species formed that differ significantly in polymerization activity. The dominant species (61 mol% of the catalytically active mixture) produced a polymer with chain properties similar to that of **9^Y^** [24], while the second active species (39 mol/%) was much more active, converting over five times more monomer. As the polymer yield reached only 71% within 1 hour of polymerization time (Table 8, run 43), it can be stated that none of these results can be due to a lack of monomer accessibility.

## 3. Materials and Methods

### 3.1. General Remarks

All operations were performed with rigorous exclusion of air and water, using standard Schlenk, high-vacuum, and argon glovebox techniques (MBraun MB 200B; < 1 ppm O_2_, < 1 ppm H_2_O, Garching, Germany). *n*-Hexane, toluene, and THF were purified by using Grubbs-type columns (MBraun SPS-800, solvent purification system) and stored inside a glovebox. CDCl_3_ was purchased from Sigma-Aldrich (St. Louis, MO, USA) and used as received. Diethyl ether and tert-butyl methyl ether (TBME) were purchased from Sigma-Aldrich, allowed to pump-freeze/thaw cycles and stored over molecular sieves (4 Å) prior to use. 1,2,3,4,5-Pentamethycyclopentadiene was purchased from Sigma-Aldrich and used as received. Isoprene was obtained from Sigma-Aldrich, dried over trioctylaluminum (Sigma-Aldrich), and vacuum transferred prior to use. 1,3-Butadiene and ethylene, as well as nitrogen and helium (atmosphere gasses for DSC), were purchased from Westphalen Gas (Münster, Germany) and purified and dried over Grubbs-type columns (MBraun) prior to use. Their amounts were determined using a Bronkhorst EL Flow Select Flow Controller (Ruurlo, The Netherlands) [Ph_3_C][B(C_6_F_5_)_4_] (**A**), [PhNMe_2_H][B(C_6_F_5_)_4_] (**B**), and B(C_6_F_5_)_3_ (**C**) were purchased from Boulder Scientific Company (Longmont, CO, USA) and used without any further purification. Precursors [Ln(AlMe_4_)_3_] (Ln = Ho, Dy, Tb, Gd) [31,32,33,34,35,36,37] and [Cp*Ln(AlMe_4_)_2_] (Ln = La, Y) were synthesized according to literature methods [12,13,14,38,39,40,41,42,43]. The NMR spectra of the precatalysts and for polymer microstructure determination were recorded at 26 °C on a Bruker AVII+ 400 Avance (^1^H: 400.11 MHz; ^13^C: 100.61 MHz)(Billerica, MA, USA). ^1^H and ^13^C shifts are referenced to internal solvent resonances and reported in parts per million (ppm) relative to tetramethylsilane (TMS). Elemental analyses were performed on an Elementar Vario MICRO cube (Hanau, Germany). IR Spectra were recorded with a NICOLET 6700 FTIR spectrometer (Thermo Fisher Scientific, Waltham, MA, USA) using a DRIFT chamber with dry KBr (Sigma-Aldrich)/sample mixtures and KBr windows. The collected data were converted using the Kubelka-Munk refinement. The molar masses (*M_W_* and *M_n_*) of the polymers were determined by size-exclusion chromatography (SEC). Sample solutions (1.0 mg polymer per mL THF) were filtered through a 0.45 µm syringe filter (PTFE, Machery-Nagel, Düren, Germany) prior to injection. SEC was performed with a pump supplied by Viscotek (GPCmax VE 2001, Malvern, UK), employing ViscoGEL columns. Signals were detected by means of a triple detection array (TDA 305) and calibrated against polystyrene standards (*M_W_*/*M_n_* < 1.15, Malvern, UK and PSS Polymer Standards Service GmbH, Mainz, Germany). The flowrate was set to 1.0 mL min^−1^. The microstructures of the polydienes were examined by means of ^1^H and ^13^C NMR experiments on the AVII+ 400 Avance spectrometer at ambient temperature, using CDCl_3_ as a solvent. Glass transition temperatures (*T_g_*) of the polymers were obtained applying a Perkin-Elmer DSC 8000 (Waltham, MA, USA) calibrated with indium and cyclohexane standards, scanning from −100 °C to +100 °C with heating rates of 20 K/min and cooling rates of 60 K/min in N_2_ atmosphere.

Single crystals for X-ray structure analysis were selected in a glovebox, coated with Parabar 10312 (previously known as Paratone N, Hampton Research, Aliso Viejo, CA, USA) and fixed on a MiTeGen MicroLoop. Data were collected on a Bruker APEX DUO instrument equipped with an IµS microfocus sealed tube and QUAZAR optics for MoK_α_ radiation (λ = 0.71073 Å). The data collection strategy was determined using COSMO [58] employing ω- and ϕ scans. Raw data were processed using APEX [59] and SAINT [60] corrections for absorption effects were applied using SADABS [61]. The structures were solved by direct methods and refined against all data by full-matrix least-squares methods on F^2^ using SHELXS, SHELXT and SHELXTL [62,63] and ShelXle [64]. All structures were refined anisotropically. For **4^Gd^**, **2^Tb^**, **2^Ho^** and **3a^Gd^** restraints were necessary to handle the disorder in a reliable manner (RIGU/SIMU and the program DSR [65]). Graphics were produced employing ORTEP-3 [66] and POV-Ray [67]. Tables S1–S5, cif, and CCDC 1951854–1951869 contain the supplementary crystallographic data for this paper. These data can be obtained free of charge via www.ccdc.cam.ac.uk/data_request/cif, or by emailing data_request@ccdc.cam.ac.uk, or by contacting The Cambridge Crystallographic Data Centre, 12 Union Road, Cambridge CB2 1EZ, UK, fax: +44 1223 336033.

### 3.2. Synthesis of [Cp*Ho(AlMe_4_)_2_] (**1^Ho^**)

According to the literature [12,13,14,38,39,40,41,42,43], in a glovebox, 500 mg (1.17 mmol) of [Ho(AlMe_4_)_3_] were dissolved in 12 mL of toluene and 1,2,3,4,5-pentamethycyclopentadiene (161 mg, 191 µL, 1.17 mmol) was added. The reaction mixture was stirred overnight, and the toluene was evaporated. The product was recrystallized from *n*-hexane at −35 °C and obtained in quantitative yield. ^1^H NMR (400 MHz, C_6_D_6_, 25 °C): *δ* = −19.4 ppm (s, C_5_(C*H*_3_)_5_), -122 ppm (br, Al(C*H*_3_)_4_). IR (Nujol, cm^−1^): 3000−2800 s (Nujol), 2726 w, 2483 w, 1463 s (Nujol), 1380 s (Nujol), 1303 w, 1235 w, 1199 w, 1023 w, 971 w, 852 w, 723 m (Nujol), 692 m (Nujol), 588 w, 547 w, 516 w. Elemental analysis (%) for C_18_H_39_Al_2_Ho (474.4 g/mol): calculated: C 45.57, H 8.29; found: C 45.62, H 8.15.

### 3.3. Synthesis of [Cp*Dy(AlMe_4_)_2_] (**1^Dy^**)

According to the literature [12,13,14,38,39,40,41,42,43], in a glovebox, 500 mg (1.18 mmol) of [Dy(AlMe_4_)_3_] were dissolved in 12 mL of toluene and 1,2,3,4,5-pentamethycyclopentadiene (162 mg, 193 µL, 1.18 mmol) was added. The reaction mixture was stirred overnight, and the toluene was evaporated. The product was recrystallized from *n*-hexane at −35 °C and obtained in quantitative yield. ^1^H NMR (400 MHz, C_6_D_6_, 25 °C): *δ* = −25.5 ppm (s, C_5_(C*H*_3_)_5_), −178 ppm (br, Al(C*H*_3_)_4_). Elemental analysis (%) for C_18_H_39_Al_2_Dy (472.0 g_/_mol): calculated: C 45.81, H 8.33; found: C 45.70, H 8.43.

### 3.4. Synthesis of [Cp*Tb(AlMe_4_)_2_] (**1^Tb^**)

In analogy to literature procedures [12,13,14,38,39,40,41,42,43], in a glovebox, 500 mg (1.19 mmol) of [Tb(AlMe_4_)_3_] were dissolved in 12 mL of toluene and 1,2,3,4,5-pentamethycyclopentadiene (163 mg, 194 µL, 1.19 mmol) was added. The reaction mixture was stirred overnight, and the toluene was evaporated. The product was recrystallized from *n*-hexane at −35 °C and obtained in quantitative yield. Elemental analysis (%) for C_18_H_39_Al_2_Tb (469.4 g/mol): calculated: C 46.16, H 8.39; found: C 46.09, H 8.52.

### 3.5. Synthesis of [Cp*Gd(AlMe_4_)_2_] (**1^Gd^**)

In analogy to literature procedures [12,13,14,38,39,40,41,42,43], in a glovebox, 500 mg (1.19 mmol) of [Gd(AlMe_4_)_3_] were dissolved in 12 mL of toluene and 1,2,3,4,5-pentamethycyclopentadiene (163 mg, 194 µL, 1.19 mmol) was added. The reaction mixture was stirred overnight, and the toluene was evaporated. The product was recrystallized from *n*-hexane at −35 °C and obtained in quantitative yield. Elemental analysis (%) for C_18_H_39_Al_2_Gd (466.7 g/mol): calculated: C 46.32, H 8.42; found: C 46.45, H 8.25.

### 3.6. Synthesis of [Cp*HoMe_2_]_3_ (**2^Ho^**)

Following the procedure published previously [23,24], complex **1^Ho^** (474 mg, 1.00 mmol) was dissolved in *n*-hexane (5 mL) and diethyl ether (148 mg, 2.00 mmol) was added under vigorous stirring. The light pink (in artificial light) and bright yellow (in sunlight) precipitate was separated via centrifugation and washed three times with *n*-hexane. Drying in vacuo yielded **2^Ho^** (330 mg, >99%) as a powder. Single crystals were obtained through recrystallization from a solution in a 1/20 mixture (mol/mol) of toluene in *n*-hexane at −35 °C. Elemental analysis (%) for C_36_H_63_Ho (990.2 g/mol): calculated: C 43.65, H 6.41; found: C 43.34, H 6.17.

### 3.7. Synthesis of [Cp*DyMe_2_]_3_ (**2^Dy^**)

Following the procedure published previously [23,24], complex **1^Dy^** (472 mg, 1.00 mmol) was dissolved in *n*-hexane (5 mL) and diethyl ether (148 mg, 2.00 mmol) was added under vigorous stirring. The acquired white precipitate was separated via centrifugation and washed three times with *n*-hexane. Drying in vacuo yielded **2^Dy^** as a powder (62 mg, >19%). Single crystals were obtained through recrystallization from a solution in a 1/20 mixture (mol/mol) of toluene in *n*-hexane at −35 °C. Elemental analysis (%) for C_36_H_63_Dy (983.4 g/mol): calculated: C 43.97, H 6.46; found: C 43.68, H 7.08.

### 3.8. Synthesis of [Cp*TbMe_2_]_3_ (**2^Tb^**) and [(Cp*Tb)_5_(CH_2_)(Me)_8_] (**3^Tb^**)

Following the procedure published previously [23,24], complex **1^Tb^** (468 mg, 1.00 mmol) was dissolved in *n*-hexane (5 mL) and diethyl ether (148 mg, 2.00 mmol) was added under vigorous stirring. The formed brownish precipitate was separated via centrifugation and washed three times with *n*-hexane. Drying the precipitate in vacuo yielded **2^Tb^** as a powder (6.5 mg, >2%). Single crystals of **2^Tb^** were obtained through recrystallization from a solution in a 1/20 mixture (mol/mol) of toluene in *n*-hexane at −35 °C. The residue was dried in vacuo as well, and an amorphous solid was obtained, recrystallization from toluene (as the dissolving in pure *n*-hexane and the mixture with toluene in 1/20 (mol/mol) failed) yielded few single crystals of **3^Tb^**. IR of **2^Tb^**(cm^−1^): 2960 s, 2940 s, 2923 s, 2903 s, 2861 s, 2723 m, 1440 s, 1434 s, 1376 s, 1180 m, 1022 m, 604 s, 597 s, 574 m, 542 m. Elemental analysis (%) for C_36_H_63_Tb (972.7 g/mol) (**2^Tb^**): calculated: C 44.45, H 6.53; found: C 45.01, H 6.50. Elemental analysis (%) for C_59_H_96_Tb_5_ (1604.1 g/mol) (**3^Tb^**): Calculated: C 44.18, H 6.28; found: C 43.47, H 6.15. The carbon value was found to be slightly out of range for both compounds, which is most likely due to increased measurement errors reasoned in the low quantity of samples.

### 3.9. Synthesis of [(Cp*Gd)_5_(CH_2_)(Me)_8_] (**3^Gd^**) and [(Cp*Gd)_4_((Et_2_O)Gd)(CH)(Me)_8_] (**3a^Gd^**)

Following the procedure published previously [23,24], complex **1^Gd^** (117 mg, 0.25 mmol) was dissolved in *n*-hexane (5 mL) and diethyl ether (148 mg, 2.00 mmol) was added under vigorous stirring at ambient temperature. After 10 min, the solvent was removed in vacuo and the apricot solid residue was recrystallized from *n*-hexane. Two different types of single crystals were obtained at −35 °C. The by far larger amount was identified as [(Cp*Gd)_5_(CH_2_)(Me)_8_] (**3^Gd^**), the side product as [(Cp*Gd)_4_((Et_2_O)Gd)(CH)(Me)_8_] (**3a^Gd^**), by means of single crystal X-ray diffractometry.

### 3.10. Synthesis of [Cp*_2_Tb(AlMe_4_)]_2_ (**4^Tb^**)

In analogy to the procedure published previously [23,24], complex **1^Tb^** (117 mg, 0.25 mmol) was dissolved in *n*-hexane (5 mL) and TBME (176 mg, 2.00 mmol) was added under vigorous stirring. After 12 h at ambient temperature, the solvent was removed in vacuo and the brown solid was recrystallized from *n*-hexane. Single crystals of **4^Tb^** were obtained at −35 °C.

### 3.11. Synthesis of [Cp*_2_Gd(AlMe_4_)]_2_ (**4^Gd^**) According to Two Different Routes

Route 1: in analogy to the procedure published previously [23,24], complex **1^Gd^** (117 mg, 0.25 mmol) was dissolved in *n*-hexane (5 mL) and TBME (176 mg, 2.00 mmol) was added under vigorous stirring. After 12 h at ambient temperature, the solvent was removed in vacuo and the obtained apricot solid residue was recrystallized from *n*-hexane. Single crystals of **4^Gd^** were obtained at −35 °C. Route 2: Following the procedure published previously [23,24], complex **1^Gd^** (117 mg, 0.25 mmol) was dissolved in precooled (−40 °C) *n*-hexane (5 mL) and precooled (−40 °C) diethyl ether (148 mg, 2.00 mmol) was added under vigorous stirring. After 10 min at −40 °C, the solvent was removed in vacuo and the obtained apricot solid residue was recrystallized from *n*-hexane. Single crystals of **4^Gd^** were obtained at −35 °C.

### 3.12. Synthesis of [(Cp*Y)_5_(Me)_6_(CH_2_)_2_] (**5^Y^**)

In a J. Young NMR tube, 16.6 mg of [Cp*YMe_2_]_3_ (**2^Y^**, 0.022 mmol) were layered with a toluene solution of 26.6 mg of **4^La^** (0.027 mmol) or 11.2 mg of **2^Y^** (0.015 mmol) were reacted with 19.7 mg of **4^Y^** (0.022 mmol). In both cases, after 1 d at ambient temperature, the originally colorless solution turned yellow and the formation of colorless crystals was observed. The crystals were suitable for X-ray diffraction and identified as **5^Y^**.

### 3.13. Synthesis of [{(Cp*Gd)_2_(Me)(CH_2_OtBu)_2_(AlMe_4_)}_2_]_2_ (**6^Gd^**)

In analogy to the procedure published previously [23,24], complex **1^Gd^** (117 mg, 0.25 mmol) was dissolved in precooled (−40 °C) *n*-hexane (5 mL) and precooled (−40 °C) TBME (176 mg, 2.00 mmol) was added under vigorous stirring. After 12 h at −40 °C few single crystals (<5% total yield) suitable for X-ray diffraction were obtained, revealing the formation of **6^Gd^**.

### 3.14. Synthesis of [Cp*La(AlMe_4_){Me_3_Al(CH_2_)OtBu}] (**7^La^**)

In analogy to the procedure published previously [23,24] complex **1^La^** (112 mg, 0.25 mmol) was dissolved in precooled (−40 °C) *n*-hexane (5 mL) and precooled (−40 °C) TBME (176 mg, 2.00 mmol) was added under vigorous stirring. After 12 h, at −40 °C, a few single crystals (< 5% total yield) suitable for X-ray diffraction were obtained, revealing the formation of **7^La^**.

### 3.15. Synthesis of [Cp*Y(AlMe_4_)(Me_3_AlOtBu)] (**8^Y^**)

In analogy to the procedure published previously [23,24], complex **1^Y^** (100 mg, 0.25 mmol) was dissolved in precooled (−40 °C) *n*-hexane (5 mL) and precooled (−40 °C) TBME (176 mg, 2.00 mmol) was added under vigorous stirring. After 12 h, at (−40 °C), few single crystals (<5% total yield) suitable for X-ray diffraction were obtained, revealing the formation of **8^Y^**.

### 3.16. Synthesis of [(Cp*Ho)_3_Me_4_(CH_2_)(thf)_2_] (**9^Ho^**)

In analogy to the synthesis of **9^Y^** published recently [24], complex **2^Ho^** (40 mg, 0.04 mmol) was dissolved in 10 mL of THF. Instant gas formation was observed. The clear solution was layered with toluene and stored at −30 °C. Crystals suitable for X-ray diffraction were obtained after 14 d and were identified as **9^Ho^**. Elemental analysis (%) for C_43_H_75_Ho_3_O_2_ (1118.8 g/mol): calculated: C 46.16, H 6.76; found: 45.87, H 6.79.

### 3.17. Polymerization of Isoprene

A detailed polymerization procedure is described as a typical example (Table 2, run 7). [Ph_3_C][B(C_6_F_5_)_4_] (**A**) (18.2 mg, 0.02 mmol) was added to a solution of **1^Gd^** (9.3 mg, 0.02 mmol) in toluene (8 mL) and the mixture was aged at ambient temperature for 30 min. Upon addition of isoprene (1.36 g, 20 mmol), the polymerization was carried out at 25 °C for 1 h. The reaction was terminated by pouring the polymerization mixture into 25 mL of methanol containing 0.1% (*w*/*w*) 2,6-di-tert-butyl-4-methylphenol as a stabilizer and stirred for 1 h. The polymer was washed with methanol and dried under vacuum at ambient temperature to constant weight.

### 3.18. Polymerization of 1,3-Butadiene

A detailed polymerization procedure is described as a typical example (Table 4, run 27). [Ph_3_C][B(C_6_F_5_)_4_] (**A**) (18.2 mg, 0.02 mmol) was added to a solution of **2^Dy^** (6.56mg, 0.007 mmol, 0.02 mmol of Dy centers) in toluene (28 mL) and the mixture was aged at ambient temperature for 30 min. The mixture was poured into an evacuated 50mL Büchi miniclave (Büchi AG, Uster, Switzerland) and 1440 mLn (60 mmol) of 1,3-butadiene were added at 90 mLn/min under constant stirring at 100 rpm. The polymerization was carried out at 25 °C for 1 h. After release of the remaining monomer pressure, if any, the reaction was terminated by pouring the polymerization mixture into 200 mL of methanol containing 0.1% (*w*/*w*) 2,6-di-tert-butyl-4-methylphenol as a stabilizer and stirred for 1 h. The polymer was washed with methanol and dried under vacuum at ambient temperature to constant weight.

## 4. Conclusions

The solvent-free pentamethylcyclopentadienyl-supported rare-earth-metal dimethyl trimers [Cp*LnMe_2_]_3_ are accessible from the monomeric [Cp*Ln(AlMe_4_)_2_] via an ether cleavage reaction for an ion size of Lu^3+^ ≤ Tb^3+^. The yields of these syntheses decrease with increasing ion size, as ligand redistribution and C-H-bond activation reactions become more favored with larger ions. The ligand redistribution pathway leads to the metallocene alkylaluminate dimers [Cp*_2_Ln(AlMe_4_)]_2_, while the C-H-bond activation results in the build-up of various multimetallic species. In case of terbium and gadolinium, square pyramidal shaped clusters of the type [(Cp*Ln)_5_(CH_2_)(Me)_8_] could be crystallographically identified. Utilization of tert-butyl methyl ether, instead of the frequently applied diethyl ether, was the only procedure that led to the formation of the dimethyl trimer in the terbium case. It was also revealed that this very ether engages in alternative activation pathways such as ether deprotonation and ether cleavage. Several of such “degradation” products could be crystallographically authenticated, including the 24-membered macrocycle [(Cp*Gd)_2_(Me)(CH_2_O*t*Bu)_2_(AlMe_4_)]_4_ or the discrete complexes [Cp*La(AlMe_4_){Me_3_Al(CH_2_)O*t*Bu}] and [Cp*Y(AlMe_4_)(Me_3_AlO*t*Bu)].

The polyisoprenes obtained from cationized [Cp*Ln(AlMe_4_)_2_] (Ln = Ho, Dy, Tb, Gd) follow the trends known from other representatives of this motif, displaying the highest trans-1,4 contents for the largest Gd(III) center (87%, PDI = 1.30). In contrast, the fabrication of high-cis-1,4 polybutadiene (97%) with the “aluminum-free” binary system [Cp*DyMe_2_]_3_/[Ph_3_C][B(C_6_F_5_)_4_] suggests that the presence of AlMe_3_ exerts a trans-directing effect in methylaluminate-based catalysts derived from mixtures [Cp*Ln(AlMe_4_)_2_]/borate. The cationization of pre-isolated cluster [(Cp*Ho)_3_Me_4_(CH_2_)(thf)_2_] accomplished polyisoprenes that support the hypothesis of multimetallic species as the active catalyst.

## Figures and Tables

**Table 1:** Comparison of the crystal structures of the Cp* rare-earth-metal bis(tetramethylaluminate) species **1^Ln^**.

Ln	ø(Ln–C^Al^)^a^	Ln–Ct	ø(∠ Ct–Ln–C^Al^)^a^	Ln---C14^ a^	ø(∠ C^Al1^–Ln–C^Al1^)^a^	ø(∠ C^Al1^–Ln–C^Al2^)^a^	T1^a^	T2^a^
	(Å)	(Å)	(°)	(Å)	(°)	(°)	(°)	(°)
La^b^	2.749(4)	2.503	106.9	3.140	75.7(1)	94.4(1)	2.2	32.5
Sm^c^	2.668(2)	2.404	106.9	3.113(2)	78.19(6)	91.41(6)	3.5	31.3
Gd	2.637(2)	2.376	107.4	3.163(2)	79.59(7)	90.05(7)	4.1	30.7
Tb	2.625(2)	2.362	107.4	3.169(2)	80.15(7)	89.27(7)	4.4	30.6
Dy	2.608(2)	2.344	107.7	3.186(2)	80.46(5)	88.78(6)	4.7	30.2
Ho	2.602(4)	2.334	108.1	3.248(4)	80.8(1)	88.0(1)	5.1	29.9
Y^d^	2.601(3)	2.340	108.6	3.304	80.5(1)	87.7(1)	5.4	29.4
Yb^c^	2.563(2)	2.293	109.1	3.354(2)	81.92(5)	85.63(5)	6.1	28.9
Lu^e^	2.545(3)	2.286	109.6	3.447	82.2(1)	84.7(1)	6.4	28.3

^a^ C^Al^ = carbon atom of a bridging methyl unit of an alkylaluminato moiety (e.g., C11, C12, C15, C16). C14 = far methyl carbon atom of the tilt alkylaluminato moiety. T1 = torsion angle C^Al1^–Ln–C^Al1^–Al1, T2 = torsion angle C^Al2^–Ln–C^Al2^–Al2. ^b^ Ref. [39]. ^c^ Ref. [43]. ^d^ Ref. [40]. ^e^ Ref. [11].

**Table 2:** Isoprene homopolymerization by [Cp*Ln(AlMe_4_)_2_] (**1^Ln^**).

Run ^a^	Precata-lyst	Cocata-lyst ^b^	*T* ^c^	*T* ^c^	Yield ^d^	cis-1,4 ^e^	trans-1,4 ^e^	3,4^e^	*M_n_* ^f^	PDI^f^	*T*_g_^g^
			(°C)	(h)	(%)	(%)	(%)	(%)	(10^4^ g·mol^−1^)		(°C)
1^i^	**1^La^**	**A**	40	24	>99	4	87	9	7	1.28	n.d.^k^
2^i^	**1^La^**	**B**	40	24	>99	3	80	17	6	1.22	n.d.^k^
3^i^	**1^La^**	**C**	40	24	>99	0	99.5	0.5	24	1.18	n.d.^k^
4^i^	**1^Nd^**	**A**	40	24	>99	14	70	16	3	2.87	n.d.^k^
5^i^	**1^Nd^**	**B**	40	24	>99	7	80	13	4	1.16	n.d.^k^
6^i^	**1^Nd^**	**C**	40	24	>99	4	92	4	13	1.35	n.d.^k^
7	**1^Gd^**	**A**	25	1	93	16	68	16	10.6	1.33	−57
8	**1^Gd^**	**B**	25	1	87	22	45	33	4.3	1.19	−43
9	**1^Gd^**	**C**	25	1	traces ^h^	- ^h^	- ^h^	- ^h^	- ^h^	- ^h^	- ^h^
10	**1^Gd^**	**C**	25	24	94	6	87	7	19.4	1.30	−64
11	**1^Tb^**	**A**	25	1	89	15	66	19	9.9	1.53	−49
12	**1^Tb^**	**B**	25	1	82	25	34	41	3.5	1.58	−53
13	**1^Tb^**	**C**	25	1	33	32	62	6	7.8	1.87	−62
14	**1^Dy^**	**A**	25	1	94	20	43	37	12.7	1.31	−52
15	**1^Dy^**	**B**	25	1	83	14	63	23	5.0	1.28	−43
16	**1^Dy^**	**C**	25	1	11	39	52	9	3.7	1.54	−65
17	**1^Ho^**	**A**	25	1	54	23	50	27	11.0	1.50	−38
18	**1^Ho^**	**B**	25	1	81	29	24	47	4.1	1.48	−23
19	**1^Ho^**	**C**	25	1	traces ^h^	- ^h^	- ^h^	- ^h^	- ^h^	- ^h^	- ^h^
20	**1^Ho^**	**C**	25	24	85	9	79	12	6.6	1.62	−62
21^i^	**1^Y^**	**A**	40	24	>99	61	21	19	2	8.95	n.d.^k^
22^i^	**1^Y^**	**B**	40	24	>99	44	29	28	6	1.59	n.d.^k^
23^i^	**1^Y^**	**C**	40	24	>99	2	94	4	9	1.59	n.d.^k^
24^j^	**1^Lu^**	**A**	40	0.25	>99	74	20	6	10	1.49	n.d.^k^
25^j^	**1^Lu^**	**B**	40	0.25	>99	70	20	10	9.5	1.48	n.d.^k^
26^j^	**1^Lu^**	**C**	40	0.25	>99	74	21	5	11.0	1.39	n.d.^k^

^a^ Conditions: 20 μmol of precatalyst, 20 μmol of cocatalyst, 20 mmol of isoprene, 8 mL of toluene. ^b^ **A** = [Ph_3_C][B(C_6_F_5_)_4_], **B** = [PhNHMe_2_][B(C_6_F_5_)_4_], **C** = B(C_6_F_5_)_3_. ^c^ *T* = polymerization temperature, *t* = reaction time. ^d^ Yield of isolated polymer. ^e^ Determined by ^1^H and ^13^C NMR spectroscopy. ^f^ Polydispersity Index (PDI); determined by size exclusion chromatography (SEC) in THF at 35 °C. ^g^ Determined by differential scanning calorimetry (DSC); heating rate: 20 K/min, cooling rate: 60 K/min. ^h^ Evaluation impossible due to low yield. ^i^ Ref. [40]; ^j^ Ref. [24]. ^k^ n.d.: not determined.

**Table 3:** Comparison of the crystal structures of the Cp* rare-earth metal dimethyl trimeric species **2^Ln^**.

Ln	Ln–C11	Ln–C12	Ln–Ct	ø (∠ Ct–Ln–C11/C12)	∠ C11–Ln–C12	∠ C11–Ln–C11’’/∠ C12–Ln–C12’’
	(Å)	(Å)	(Å)	(°)	(°)	(°)
Tb	2.54(2)	2.57(2)	2.389	114.0	79.8(3)	83(1)/83.9(7)
Dy	2.63(3)	2.49(3)	2.360	111.6	81(1)	85.1(9)/79.9(3)
Ho	2.58(2)	2.58(2)	2.399	112.7	80.7(3)	77.5(7)/87.2(8)
Y ^a^	2.539	- ^a^	2.359	112.9	80.0	82.8 ^a^
Lu ^b^	2.45(2)	2.53(3)	2.317	111.9	80.4(3)	88(1)/79 (1)

^a^ Ref. [23], centrosymmetric space group. ^b^ Ref. [24].

**Table 4:** 1,3-Butadiene homopolymerization by [Cp*LnMe_2_]_3_ (**2^Ln^**).

Run ^a^	Precata-lyst	Cocata-lyst ^b^	*T* ^c^	*t* ^c^	Yield ^d^	cis-1,4 ^e^	trans-1,4 ^e^	1,2 ^e^	*M_n_* ^f^	PDI ^f^	*T*_g_^g^
			(°C)	(h)	(%)	(%)	(%)	(%)	(10^4^ g·mol^−1^)		(°C)
27	**2** ^**Dy**^	**A**	25	60	99	97	0	3	16.2	1.63	−102
28	**2** ^**Dy**^	**B**	25	60	92	96	0	4	20.4	1.64	−102
29	**2** ^**Ho**^	**A**	25	60	90	91	4	5	5.5	1.52	−102
30	**2** ^**Ho**^	**B**	25	60	59	94	2	4	32.8	1.35	−101
31^j^	**2** ^**Y**^	**A**	25	60	21	91	4	5	76	1.58	−97
32^j^	**2** ^**Y**^	**B**	25	60	48	95	2	3	50	1.20	−94
33^j^	**2** ^**Lu**^	**A**	25	60	>99	85	8	7	40	1.84	−94
34^j^	**2** ^**Lu**^	**B**	25	60	95	85	8	7	33	1.77	−93

^a^ Conditions: 20 μmol of precatalyst, 20 μmol of cocatalyst, 1440 mLn of 1,3-butadiene, 8 mL of toluene. ^b^ **A** = [Ph_3_C][B(C_6_F_5_)_4_], **B** = [PhNHMe_2_][B(C_6_F_5_)_4_]. ^c^ *T* = polymerization temperature, *t* = reaction time. ^d^ Yield of the isolated polymer. ^e^ Determined by ^1^H and ^13^C NMR spectroscopy. ^f^ Determined by SEC in THF at 35 °C. ^g^ Determined on a DSC 8000; heating rate: 20 K/min, cooling rate: 60 K/min. ^j^ Ref. [24].

**Table 5:** Isoprene homopolymerization by [Cp*LnMe_2_]_3_ (**2^Ln^**).

Run^a^	Precata-lyst	Cocata-lyst ^b^	*T* ^c^	*t* ^c^	Yield ^d^	cis-1,4 ^e^	trans-1,4 ^e^	3,4 ^e^	*M_n_* ^f^	PDI ^f^	*T*_g_^g^
			(°C)	(h)	(%)	(%)	(%)	(%)	(10^4^ g·mol^−1^)		(°C)
35	**2** ^**Dy**^	**A**	25	60	90	53	13	34	6.3	3.15	−36
36	**2** ^**Dy**^	**B**	25	60	89	33	25	43	14.6	1.38	−24
37	**2** ^**Ho**^	**A**	25	60	96	36	18	46	15.5	1.27	−20
38	**2** ^**Ho**^	**B**	25	60	87	33	16	51	12.0	1.13	−20
39^j^	**2** ^**Y**^	**A**	25	60	>99	51	17	32	8.4	2.33	−47
40^j^	**2** ^**Y**^	**B**	25	60	90	36	11	53	14.1	1.16	−27
41 ^j^	**2** ^**Lu**^	**A**	25	60	>99	21	21	58	26.8	2.00	−16
42 ^j^	**2** ^**Lu**^	**B**	25	60	>99	21	22	57	29.2	1.38	−17

^a^ Conditions: 20 μmol of precatalyst, 20 μmol of cocatalyst, 20 mmol of isoprene, 8 mL of toluene. ^b^ **A** = [Ph_3_C][B(C_6_F_5_)_4_], **B** = [PhNHMe_2_][B(C_6_F_5_)_4_]. ^c^ *T* = polymerization temperature, *t* = reaction time. ^d^ Yield of the isolated polymer. ^e^ Determined by ^1^H and ^13^C NMR spectroscopy. ^f^ Determined by SEC in THF at 35 °C. ^g^ Determined on a Perkin Elmer DSC 8000; heating rate: 20 K/min, cooling rate: 60 K/min. ^j^ Ref. [24].

**Table 6:** Comparison of selected metrical data of known [Cp*_2_LnAlMe_4_)]_2_ (**4^Ln^**).

Ln	ø(Ln–C^Al^) ^a^	Ln–Ct1	Ln–Ct2	ø(∠ Ct–Ln–C^Al^) ^a^	∠ Ct1–Ln–Ct2	∠ C^Al1^–Ln–C^Al1’^
	(Å)	(Å)	(Å)	(°)	(°)	(°)
La ^b^	2.848(2)	2.522	2.516	104.5	139.62	87.36(4)
La(Tol) ^b,c^	2.867(4)	2.527	2.513	104.3	140.04	87.7(1)
Sm^d^	2.75(2)	2.414	2.419	105.1	138.6	85.0(5)
Sm(Tol) ^c,e^	2.749(4)	2.421	2.424	105.8	135.7	87.7(1)
Gd	2.712(2)	2.399	2.400	104.9	138.5	86.95(6)
Tb	2.690(2)	2.385	2.380	104.4	139.7	86.9
Y ^f^	2.66	n.d.	n.d.	n.d.	n.d.	84.9(7)
Yb(Tol) ^c,e^	2.634(2)	2.314	2.326	105.0	138.1	87.12(6)

^a^ Ln(Tol) = unit cell contains one molecule of toluene; C^Al^ = carbon of a methyl unit of an alkylaluminato moiety. ^b^ Ref. [40]. ^c^ Unit cell contains one toluene molecule. ^d^ Ref. [52]. ^e^ Ref. [43]. ^f^ Ref. [51].

**Table 7:** Comparison of the pyramidal pentanuclear crystal structures of **3^Ln^**, **3a^Gd^** and **5^Y^**.

	Ln–C^Cp*^	**Ln1-4** **–Ct1-4**	**Ln5–Ct5/O**		**Ln1-4–µ_2_C**		**Ln5–µ_3_C**		**Ln1-4** **–µ_3_C**	**C01** **–Ln5**		**C01** **–Ln1-4**
	(Å)	(Å)	(Å)		(Å)		(Å)		(Å)	(Å)		(Å)
**3^Tb^**	2.701(4)–2.808(5)	2.443–2.448.	2.495		2.569(5)–2.658(6)		2.610(5)–2.657(5)		2.663(5)–2.774(5)	2.493(4)		2.462(5)–2.529(5)
**3^Gd^**	2.69(1)–2.816(5)	2.450–2.471	2.497		2.533(6)–2.571(6)		2.593(7)–2.633(6)		2.631(6)–2.677(6)	2.373(7)		2.494(7)–2.533(7)
**3a^Gd^**	2.686(8)–2.923	2.504–2.595	2.487		2.569(9)–2.629(8)		2.571(8)–2.582(8)		2.811(8)–2.898(8)	2.222(7)		2.506(7)–2.531(7)
**5^Y^**	2.64(1)–2.685(8)	2.372–2.391	-		2.494(9)–2.78(1)		2.477(8)–2.711(8)		2.399(9)–2.638(9)	-		-
	**∠** **(C01** **–** **Ln1-4** **–** **µ_2_C)**			**∠** **(C01** **–** **Ln5** **–** **µ_3_C)**		**∠** **(µ_3_Cx** **–** **Ln5µ_3_Cx+2)**		**∠** **(Ln1-4** **–** **C01** **–** **Ln5)**			**∠** **(Ln1/3** **–** **C01** **–** **Ln2/4)**	
	(°)			(°)		(°)		(°)			(°)	
**3^Tb^**	74.97(1)–78.0(2)			77.6(2)–78.1(1)		156.8(2)/155.6(2)		87.4(1)–90.5(1)			175.7(2)/176.4(2)	
**3^Gd^**	76.0(2)–78.1(2)			73.2(2)–74.2(2)		147.3(2)–147.4(2)		92.9(2)–96.2(2)			170.9(3)/171.1(3)	
**3a^Gd^**	77.3(3)–78.9(2)			85.4(3)–87.1(4)		172.5(3)/173.2(3)		89.9(3)–90.6(2)			179.3(3)/178.8(4)	
**5^Y^**	-			-		134.7(3)/135.2(4)		-			-	

**Table 8:** Isoprene homopolymerization by [(Cp*Ho)_3_Me_4_(CH_2_)(thf)_2_] (**9^Ho^**).

Run ^a^	Precata-lyst	Cocata-lyst ^b^	*T* ^c^	*t* ^c^	Yield ^d^	cis-1,4 ^e^	trans-1,4 ^e^	3,4 ^e^	*M_n_* ^f^	PDI ^f^	*T*_g_^g^
			[°C]	[h]	[%]	[%]	[%]	[%]	[10^4^ g·mol^−1^]		[°C]
43	**9** ^**Ho**^	**A**	25	1	71 ^h^	48 ^h^	16^h^	36^h^	13.2 ^h^	1.34^h^	−36^h^
44	**9** ^**Ho**^	**B**	25	1	9	74	1	25	22.0	1.60	−49
45^i^	**9** ^**Y**^	**A**	25	1	48	44	14	41	13.9^h^	1.42^h^	−31
46^i^	**9** ^**Y**^	**B**	25	1	7	76	0	24	55.1	1.97	−48

^a^ Conditions: 20 μmol of precatalyst (7 μmol, 20 μmol of Ln centers), 20 μmol of cocatalyst, 20 mmol of isoprene, 8 mL of toluene. ^b^ **A** = [Ph_3_C][B(C_6_F_5_)_4_], **B** = [PhNHMe_2_][B(C_6_F_5_)_4_]. ^c^ *T* = polymerization temperature, *t* = reaction time. ^d^ Yield of the isolated polymer. ^e^ Determined by ^1^H and ^13^C NMR spectroscopy. ^f^ Determined by GPC (SEC) in THF at 35 °C. ^g^ Determined by DSC; heating rate: 20 K/min, cooling rate: 60 K/min. ^h^ The products (runs 43 and 45) were identified as a blend of two polymers. This cannot be resolved by NMR spectroscopy or DSC, so the averages are given for the microstructure and glass transition temperature. GPC results instead are given for the main component of the blend (61 mol% (run 43) and 75 mol% (run 45), respectively). The smaller part of run 43 (39 mol%) contains chains of *M_n_* = 85.0·10^4^ g/mol and a PDI of 1.39. ^i^ Ref. [24].

## Supplementary Materials

The following are available online, Supporting Information (pdf) containing crystal data. CIF files containing crystal data for complexes **1^Ho^**, **1^Dy^**, **1^Tb^**, **1^Gd^**, **2^Ho^**, **2^Dy^**, **2^Tb^**, **3^Tb^**, **3^Gd^**, **3a^Gd^**, **4^Tb^**, **4^Gd^**, **5^Y^**, **7^La^**, **8^Y^**, **9^Ho^**. The latter data have been deposited as well at CCDC (1951854-1951869).
