# Supplementary material for: C–H-Bond Activation and Isoprene Polymerization Studies Applying Pentamethylcyclopentadienyl-Supported Rare-Earth-Metal Bis(Tetramethylaluminate) and Dimethyl Complexes
Source: Molecules. 2019 Oct 15;24(20):3703. doi: 10.3390/molecules24203703 (PMC6832758; doi:10.3390/molecules24203703)
Supplement: Supplementary file 1 [file molecules-24-03703-s001.pdf]

molecules

MDPI

Supplementary Material

C-H Bond Activation and Isoprene Polymerization Studies Applying Pentamethylcyclopentadienyl-Supported Rare-Earth-Metal Bis(Tetramethylaluminate) and Dimethyl Complexes

Christoph O. Hollfelder,[1] Georgios Spiridopoulos,[1] Daniel Werner,[1] Melanie Meermann-Zimmermann,[1,2] Karl W. Törnroos,[2] Cäcilia Maichle-Mössmer,[1] and Reiner Anwander[1,*]

Crystallographic Data

Table S1. Crystal data and structure refinement for $\mathbf{1}^{Ln}$

	$\mathbf{1}^{Ho}$	$\mathbf{1}^{Dy}$	$\mathbf{1}^{Tb}$	$\mathbf{1}^{Gd}$
CCDC	1951859	1951855	1951854	1951857
Empirical formula	$C_{18}H_{39}Al_2Ho$	$C_{18}H_{39}Al_2Dy$	$C_{18}H_{39}Al_2Tb$	$C_{18}H_{39}Al_2Gd$
M_W [g mol^{-1}]	474.38	471.95	468.37	466.70
T [K]	100(2)	100(2)	100(2)	100(2)
Wavelength [Å]	0.71073	0.71073	0.71073	0.71073
Crystal system	Orthorhombic	Orthorhombic	Orthorhombic	Orthorhombic
Space group	Pbca	Pbca	Pbca	Pbca
a [Å]	17.122(3)	17.1114(6)	17.1031(8)	17.0985(3)
b [Å]	14.301(3)	14.2885(5)	14.3081(7)	14.3377(3)
c [Å]	17.950(3)	17.9364(6)	17.9791(10)	17.9667(4)
α [°]	90	90	90	90
β [°]	90	90	90	90
γ [°]	90	90	90	90
Volume [Å^3]	4395.3(15)	4385.4(3)	4399.7(4)	4404.59(16)
Z	8	8	8	8
Density (calc) [mg mm^{-3}]	1.434	1.430	1.414	1408
Absorption coefficient	3.675	3.482	3.289	3.086
Theta range [°]	2.174 to 28.52	2.564 to 28.70	2.265 to 23.76	2.561 to 30.122
Reflections	41352	83800	37471	63637
Independent reflections	5607 (R_{int} =0.0819)	4845 (R_{int} =0.0280)	4857 (R_{int} =0.0304)	4863 (R_{int} =0.0307)
Data / Restraints / Parameters	5607 / 0 / 247	4845 / 0 / 247	4857 / 3 / 255	4863 / 0 / 313
R_1 (obs)[a]	0.0317	0.0125	0.0171	0.0165
wR2 (all)[b]	0.0834	0.0302	0.0381	0.0325

[a] Final R indices [$I > 2\sigma(I)$];

[b] R indices (all data).

Table S2. Crystal data and structure refinement for $\mathbf{2}^{Ln}$

	$\mathbf{2}^{Ho}$	$\mathbf{2}^{Dy}$	$\mathbf{2}^{Tb}$
CCDC	1951861	1951856	1951860
Empirical formula	$C_{36}H_{63}Ho_3$	$C_{36}H_{63}Dy_3$	$C_{36}H_{63}Tb_3$
M_W [g mol^{-1}]	990.65	983.36	972.62
T [K]	190(2)	100(2)	170(2)
Wavelength [Å]	0.71073	0.71073	0.71073
Crystal system	Hexagonal	Hexagonal	Hexagonal
Space group	$P6_3$	$P6_3$	$P6_3$
a [Å]	12.25400(10)	12.2235(13)	12.323(2)
b [Å]	12.25400(10)	12.2235(13)	12.323(2)
c [Å]	14.5911(2)	14.4643(16)	14.512(2)
α [°]	90	90	90
β [°]	90	90	90
γ [°]	120	120	120
Volume [Å^3]	1897.47(4)	1871.6(4)	1908.5(7)
Z	2	2	2
Density (calc) [mg mm^{-3}]	1.734	1.745	1693
Absorption coefficient	6.214	5.947	5.518
Theta range [°]	2.373 to 28.265	4.320 to 27.195	2.211 to 28.55
Reflections	14472	41262	11852
Independent reflections	3140 (R_{int} =0.0284)	4320 (R_{int} =0.0463)	3131 (R_{int} =0.0349)
Data / Restraints / Parameters	3140 / 652 / 168	2469 / 811 / 238	3131 / 622 / 238
R_1 (obs)[a]	0.0335	0.0270	0.0258
wR2 (all)[b]	0.0913	0.0596	0.0494

[a] Final R indices [$I > 2\sigma$ (I)];

[b] R indices (all data).

Table S3. Crystal data and structure refinement for $\mathbf{3^{Ln}}$ and $\mathbf{3a^{Gd}}$

	$\mathbf{3^{Tb}}$	$\mathbf{3^{Gd}}$	$\mathbf{3a^{Gd}}$
CCDC	1951862	1951858	1951863
Empirical formula	$C_{65}H_{115}Tb_5$	$C_{59}H_{100}Gd_5$	$C_{55.5}H_{101}Gd_5O$
M_W [g mol^{-1}]	1691.16	1595.63	1570.61
T [K]	100(2)	100(2)	180(2)
Wavelength [Å]	0.71073	0.71073	0.71073
Crystal system	Triclinic	Triclinic	Triclinic
Space group	$P\bar{1}$	$P\bar{1}$	$P\bar{1}$
a [Å]	13.8572(5)	13.8814(11)	13.882(2)
b [Å]	13.9346(5)	13.9407(12)	21.176(3)
c [Å]	17.9257(6)	17.4653(14)	23.125(4)
α [°]	70.9640(10)	101.1150(10)	85.953(4)
β [°]	75.233(2)	107.0640(10)	72.925(2)
γ [°]	89.4320(10)	91.6700(10)	84.468(4)
Volume [Å^3]	3153.82(19)	3157.2(4)	6461.5(18)
Z	2	2	4
Density (calc) [mg mm^{-3}]	1.781	1.678	1615
Absorption coefficient	5.569	5.210	5.091
Theta range [°]	2.211 to 27.055	2.243 to 28.755	2.940 to 26.430
Reflections	85368	57956	110580
Independent reflections	16286 (R_{int} =0.0397)	15577 (R_{int} =0.0760)	28444 (R_{int} =0.0437)
Data / Restraints / Parameters	16286 / 465 / 831	15577 / 2490 / 946	28444 / 4793 / 1451
R_1 (obs)[a]	0.0348	0.0364	0.0486
wR2 (all)[b]	0.0648	0.0876	0.1335

[a] Final R indices [$I > 2\sigma$ (I)];

[b] R indices (all data).

Table S4. Crystal data and structure refinement for $\mathbf{4^{Ln}}$ and $\mathbf{5^{Y}}$

	$\mathbf{4^{Tb}}$	$\mathbf{4^{Gd}}$	$\mathbf{5^{Y}}$
CCDC	1951866	1951868	1951869
Empirical formula	$C_{54}H_{98}Al_2Tb_2$	$C_{54}H_{98}Al_2Gd_2$	$C_{65}H_{105}Y_5$
M_W [g mol^{-1}]	1119.12	1029.61	1331.03
T [K]	100.15	190(2)	103(2)
Wavelength [Å]	0.71073	0.71073	0.71073
Crystal system	Triclinic	Monoclinic	Triclinic
Space group	$P\bar{1}$	$P2_1/n$	$P\bar{1}$
a [Å]	10.6342(2)	10.4209(2)	13.0959(4)
b [Å]	11.7105(2)	17.2205(3)	13.6813(4)
c [Å]	12.4893(2)	17.2205(3)	19.3347(6)
α [°]	70.2974(6)	90	74.7470(10)
β [°]	72.7174(7)	91.1529(11)	70.9090(10)
γ [°]	86.0505(7)	90	82.0710(10)
Volume [Å^3]	1397.30(4)	2537.44(8)	3153.21(17)
Z	1	2	2
Density (calc) [mg mm^{-3}]	1.330	1.348	1.402
Absorption coefficient	2.572	2.653	4.587
Theta range [°]	2.28 to 30.02	2.421 to 26.11	2.28 to 24.99
Reflections	53230	69851	36896
Independent reflections	8125 (R_{int} =0.0265)	6297 (R_{int} =0.0305)	11163 (R_{int} =0.0568)
Data / Restraints / Parameters	8125 / 12 / 466	6297 / 0 / 272	11163 / 1353 / 723
R_1 (obs)[a]	0.0172	0.0175	0.0675
wR2 (all)[b]	0.0411	0.0437	0.1671

[a] Final R indices [$I > 2\sigma(I)$];

[b] R indices (all data).

Table S5. Crystal data and structure refinement for 7^{La}, 8^{Y}, and 9^{Ho}

	7^{La}	8^{Y}	9^{Ho}
CCDC	1951867	1951865	1951864
Empirical formula	$C_{22}H_{47}Al_2LaO$	$C_{21}H_{45}Al_2YO$	$C_{43}H_{75}Ho_3O_2$
M_W [g mol^{-1}]	520.46	456.44	1118.82
T [K]	123(2)	100(2)	100(2)
Wavelength [Å]	0.71073	0.71073	0.71073
Crystal system	Triclinic	Monoclinic	Orthorhombic
Space group	$P\bar{1}$	$P2_1/c$	$P2_12_12_1$
a [Å]	9.433(3)	11.212(2)	12.8540(12)
b [Å]	17.696(6)	16.416(3)	14.3860(13)
c [Å]	19.017(7)	28.329(6)	22.921(2)
α [°]	109.815(8)	90	90
β [°]	102.367(7)	90.18(3)	90
γ [°]	103.789(8)	90	90
Volume [Å^3]	2745.2(17)	5214.0(19)	4238.5(7)
Z	4	8	4
Density (calc) [mg mm^{-3}]	1.259	1.163	1.753
Absorption coefficient	1.628	2.311	5.578
Theta range [°]	2.2122 to 25.5193	2.591 to 26.78	2.272 to 29.14
Reflections	80210	31101	53672
Independent reflections	13275 (R_{int} =0.1042)	11212 (R_{int} =0.0424)	11427 (R_{int} =0.0445)
Data / Restraints / Parameters	13275 / 0 / 551	11212 / 0 / 482	11427 / 0 / 493
R_1 (obs)[a]	0.0469	0.0376	0.0416
wR2 (all)[b]	0.0871	0.0814	0.0421

[a] Final R indices [$I > 2\sigma(I)$];

[b] R indices (all data).
